# Environmental dust repelling from hydrophilic/hydrophobic surfaces under sonic excitations

**DOI:** 10.1038/s41598-020-76418-2

**Published:** 2020-11-09

**Authors:** Abba Abdulhamid Abubakar, Bekir Sami Yilbas, Hussain Al-Qahtani, Ammar Alzaydi

**Affiliations:** 1 Mechanical Engineering Department, King Fahd University of Petroleum and Minerals, Dhahran, 31261 Saudi Arabia; 2 Center of Research Excellence in Renewable Energy (CoRE-RE), KFUPM, Dhahran, 31261 Saudi Arabia; 3 K.A. CARE Energy Research & Innovation Center at Dhahran, Dhahran, Saudi Arabia

**Keywords:** Mechanical engineering, Engineering

## Abstract

Dust repelling from transparent polyvinyl chloride film surface via sonic excitation is examined and dynamics of repelled (inflight) dust particles are analyzed. An experimental rig is designed and built to assess the vibrational characteristics of the polyvinyl chloride film at different frequencies of sonic excitation. A high speed recording system and tracking program are utilized monitoring and evaluating the dynamics of the inflight particles. The dynamics of inflight particles are also simulated numerically and the predictions are compared with those of the experimental data. In order to examine the influence of dust particle adhesion on the dynamics of the inflight particles, the polyvinyl chloride film surface is hydrophobized through dip coating by functionalized nano-silica particles. Improvement of the optical transmittance of the dust mitigated film is determined via outdoor tests. The findings demonstrate that sonic excitation repels the particles from the film surface and it is more pronounced at 64 Hz excitation frequency while demonstrating that sonic excitation can be used for dust removal from transparent surfaces. The mitigation via sonic excitation improves the optical transmittance of the dusty surface by 77%, which becomes more apparent for hydrophobic surfaces.

## Introduction

The mitigation of environmental dust from surfaces becomes unavoidable because of regular dust settlements on surfaces in open environments. This becomes particularly important for solar energy devices because of the prolonged duration of dust settlement significantly influences the optical properties of the device surfaces and reduces the device performance. The solar radiation reaching on to the device surfaces is immensely affected by the accumulated dust layer^1^, which is more pronounced in nearby desert environments where the frequent dust storms occur. Some cleaning techniques were used for dust mitigation, such as brushing^2^, electrostatic repelling^3,4^, water spreading^5^, air puffing^6^, liquid droplet rolling^7^, mechanical vibration^8^, ultrasonic cleaning^9^, etc. These techniques are effective in dust mitigation from surfaces even though they have some shortcomings in terms of high energy consumptions, scarcity of clean water, and expensive air compression. In addition, the operation and maintenance costs of the mechanical systems for automated brushing, air-jet blowing, and water splashing are expected to be enormously high in desert environments. The weather conditions do not appear to be encouraging for self-cleaning applications (via utilizing the mechanism of water droplet rolling) such that the rainfall, in general, is not feasible most of the time during the entire seasons in arid regions. Although electrostatic dust repelling from surfaces has promising feature, yet removal of small dust particles from surfaces becomes challenging because of strong adhesion of these particles on the surfaces. Moreover, utilizing the mechanism of the mechanical vibration towards cleaning the dusty surfaces can only be effective within limited frequency and amplitude ranges because of avoiding the structural damages of the solar energy device^10^. One of the alternative methods can involve with repelling of dust particles with acoustic radiation under the sound wave excitations. Although some studies were presented incorporating utilization of ultrasonic excitation for particle removal from surfaces, the environment incorporated is mainly concerned with the liquids^9^, which may become costly for dust mitigation from energy harvesting device surfaces. Hence, investigation of dust mitigation from optically transparent surfaces via acoustic radiation becomes essential.

Cleaning of surfaces using the acoustic waves has taken attention for the application of energy harnessing equipment^11^. The main focus was dust mitigation via increasing detachment of the dust particles from surfaces using the acoustic radiative power. The approach, that introduces the ultrasonic waves for removal of airborne particles including small size dust, pollens, and industrial low size wastes, reported to work well; however, the system could operate with the presence of a thin water layer on surfaces^12^. Although the ultrasonic cleaning method provided the effective cleaning of small particles, the scarcity of clean water in rural areas could limit the practical application of such arrangements in large scale solar energy harvesting farms. The use of sonic radiation could be extended to include fouling in tubing systems^13^ and residuals removal in manufacturing, particularly additive manufacturing system^9,12^. In any case, the use of clean water becomes a necessity for such cleaning applications. Moreover, introducing mechanical excitations on surfaces via piezoelectric actuators and mechanical accelerators could clean the surfaces in ambiances where airborne particles are high present at high concentrations in air. However, it became necessary to use the additional mechanical system(s) to remove the repelled particles from surfaces such mechanical brushes^8^ or to utilize gravitational potential, via surface inclination, to displace the repelled particles from surfaces^14^. The vibrational excitation of the antistatic coated surface could ease the mitigation of the dust particles from surfaces; however, the vibrational characteristics of the surface remained important for the efficient cleaning process. The combination of strong acoustic wave (sound wave) and mechanical vibration could be used effectively to clean the porous surfaces, which became particularly important for producing the biomedical parts^15^. However, practical applicability of such arrangements for dust mitigation is questionable because of relatively smooth surfaces involved in energy harvesting equipment. Moreover, the acoustic excitation towards removal of deposits from surfaces could be favorable for heat transfer enhancement^16^; however, the sound waves generated could disperse and propagate in all directions in the liquid while resulting in loss of wave intensity, which lowered the mitigation of deposits from surfaces. Nevertheless, sound wave excitation in air ambient for the mitigation of environmental dust needs to be explored for practical applications.

On the other hand, environmental dust contains various elements with salt and oxide compounds. Some of the slat compounds in the dust particles do not conform stoichiometric ratio while creating additional ionic charges on the particle surfaces^17^. This adds to the interfacial adhesion, due to the van der Walls forces, between the particles and the settled surfaces. In addition, the charged particles form clusters-like edifices and they can attach onto the dust particle surfaces with large sizes^18^. The combination of particle clustering and ionic forces considerably enhances the work of adhesion required for dust removal from surfaces, i.e. increased contact area at the interface on the settled surface and strong interfacial forces enhance particle adhesion. One of the methods reducing the individual and clustered particle adhesion on the settled surfaces is to reduce the contact area between the particles and the settled surfaces. This can be achieved, possible, through proper texturing of the surface^19^. The surface texture with hierarchically distributed mico/nanopillars are favorable because such texture topology gives rise to hydrophobic state on the surfaces^20^. Hydrophobizing the surface lowers the transmittance of the optically transparent samples; however, overall reduction in optical transmittance of hydrophobized surface over the visible spectrum is almost 10% of the none-hydrophobized surface^21^. In environments subjected to frequent heavy dust settlements, hydrophobizing the surfaces eases dust mitigation from surfaces; in which case, overall optical transmittance can improve. Moreover, reducing the surface free energy of the textures causes a further reduction in the adhesion of the particles on surfaces. Hydrophizing the surface having low surface free energy, via texturing and chemical modification, can ease the mechanical repelling of dust particles from the surface due to low adhesion^18^. Hence, lowering the free energy of textured surfaces provides better opportunities to mitigate the environmental dust particles surfaces via acoustic excitations in terms of sonic waves. Acoustic excitations for dust mitigation have successfully applied in the previous studies to remove soot and ashes from tune banks, particularly at high-temperature applications^22^. The low frequency and high-intensity sound waves were effectively used removing the charged particles (powders) from the collection plate of an electrostatic precipitator^22^. Although acoustic levitation of dust particles enables are swept by the airflow, the flow forming a vortex structure is required for mitigating the levitated particles from the surfaces^23,24^. However, generating such flow structures sustaining the cleaning of large areas for prolonged durations remains difficult to achieve. In addition, the particle removal under sonic excitation was studied earlier and the focus was the removal of tooth^22^ or charged particles^23^. However, dust particles have larger densities than sooth and low static charges unlike those of the cases reported in the early work^22^. Hence, the removal of dust particles from surfaces in dry environments under the sonic influence becomes interestingly new research and accomplishments of dust mitigation from surfaces by sonic excitation becomes fruitful despite the fact that the practical applications of sonic excitation for dust mitigation in: i) cleaning of sensor surfaces for autonomous systems, and ii) efficient operation of solar energy harvesting devices require further investigations. Nevertheless, the present work is proposed to investigate the dust removal from hydrophilic/hydrophobic and optically transparent polyvinyl chloride film surfaces using the sonic excitations pertinent to cleaning applications of protective layer for photovoltaic applications. The sample (polyvinyl chloride film) surfaces are hydrophobized by depositing functionalized nano-silica particles via dip coating. An experimental rig is designed and built for sonic excitations and dust removal. A high speed recording system is utilized monitoring and tracking the repelling dust particles from surfaces during the sonic excitations. An analytical approach is introduced to formulate the repelled dust particle dynamics and resulting predictions are compared with those of the experimental findings. The outdoor tests are carried out to evaluate the optical transmittance of dust repelled surfaces.

## Experimental

Polyvinyl chloride film with 0.14 mm thickness and 120 mm diameter were used as samples. The sample surfaces were hydrophobized via a dip-coating technique through depositing the functionalized nano-silica-particles. The nanoparticles were synthesized in accordance with the early work^25^. The wetting of the coated surface was determined via goniometer (Kyowa, model DM 501) as similar to the previous work^26^. A high resolution camera and software were incorporated in the goniometer to capture and analyze the contact angle. De-ionized water was used in the measurements and droplet volume was controlled with an automatic dispensing system having a volume step resolution of 0.1 µL. The sessile droplet contact angle was evaluated using the high-precision drop shape analysis (HPDSA) technique in line with the early work^26^. Hence, the contact angle of the coating surface was 152° ± 3° with hysteresis 5° ± 2°. In addition, the contact angle of as received polyvinyl chloride film was measured as 82° ± 2° with contact angle hysteresis 41° ± 3°. The coating texture topology and dust particle adhesion on hydrophobic/hydrophilic surfaces are obtained using an atomic force microscope (AFM/SPM) probe with the friction mode.

**Figure 1:**
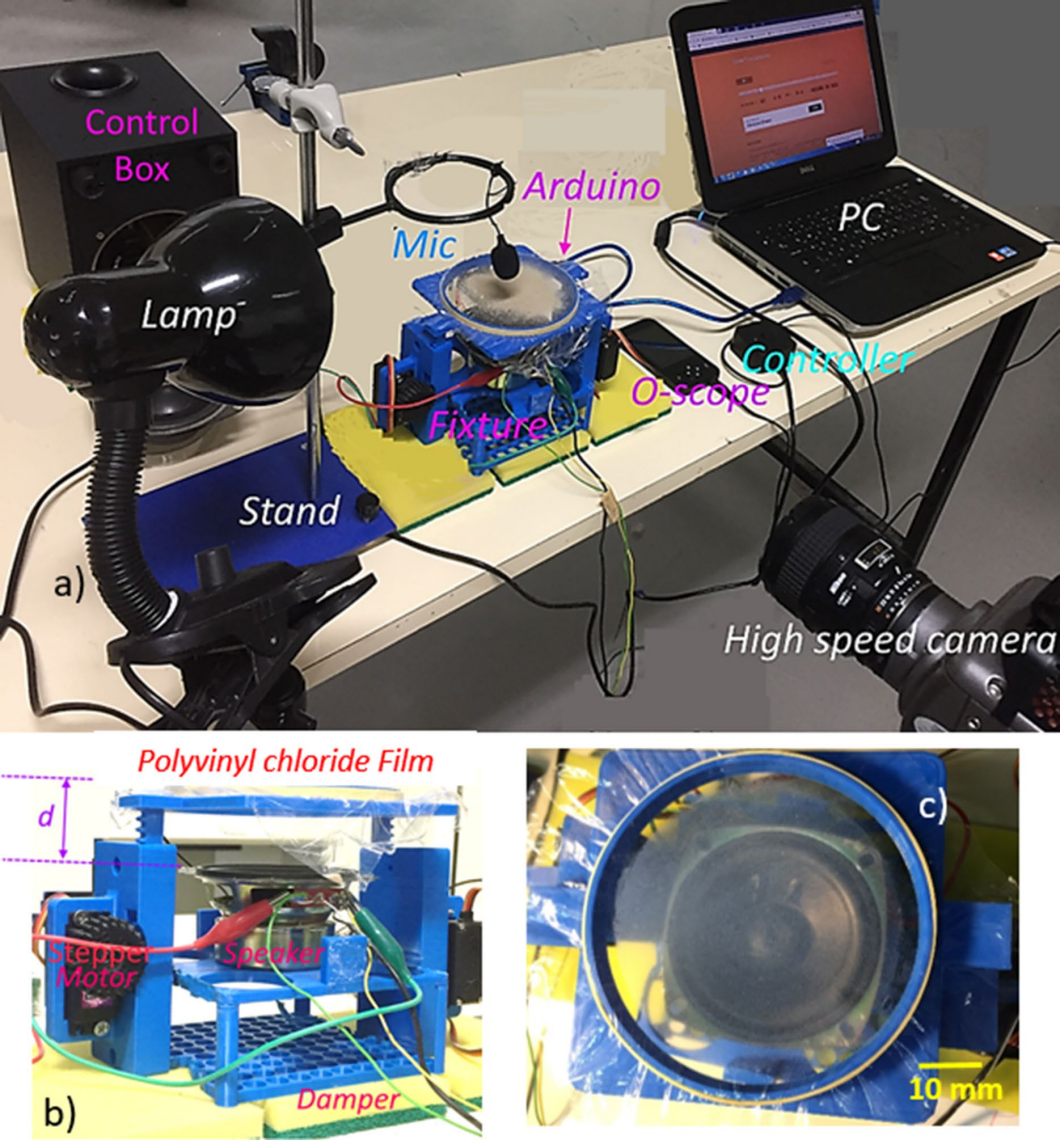
Optical image of the set-up: (**a**) complete unit, (**b**) speaker unit and d is the standoff distance, (**c**) polyvinyl chloride film surface.

A fixture with two-axes freedom was built via using a 3D printer. The fixture accommodates both the circular sample holder and the loudspeaker with foam insulation in between them. Figure 1 shows the optical image. The loudspeaker (Edifier Inc.) operating at 9 V (DC) and 0.44 A was used to generate the sound waves at various frequencies. To minimize the mechanical disturbance of the polyvinyl chloride film by the loudspeaker mechanical vibration, vibration-reducing foams were used to isolate the loudspeaker from the polyvinyl chloride film holder. An accelerometer was incorporated ensuring the frequency measurement of polyvinyl chloride film during the sonic excitation by the loudspeaker at various frequencies. Initially, many tests were conducted securing the measurement repeatability and fixture stability at various frequencies of the sonic excitations. A high-speed camera (Speed Sense 9040) was utilized monitoring film oscillations when subjected to the sonic excitations and the movement of the repelled dust particles from the film surface. In the dust particles repelling experiments, the dust layer of almost 150 µm was deposited onto the polyvinyl chloride film surface. Initially, many tests were carried out calibrating the film response to the sonic excitations and dust particles' motion in terms of vertical and lateral motions. The tracker program was used to evaluate the dust particle dynamics from the recorded data. The high-speed records were obtained at 5,000 frames-per-second (fps) at a resolution of 1280 × 800 pixels. The pixel size of the images was 14 µm × 14 µm. The repeatability of the recording tests was assessed and the standard error estimated was in the order of 3%. The uncertainty (± *u*) of the measurements was evaluated incorporating the data measured (vertical and horizontal repelling heights of the particles). The confidence level of 95% was ensured basing the data repeatability. The uncertainty (*σ*_*u*_) is^27^: ${\sigma }_{u}=\sqrt{{\int }_{{x}_{o}}^{{x}_{n}}{\left(x-{\mu }_{e}\right)}^{2}p\left(x\right)dx}$, here, *µ*_*e*_ represents the mean/expected value of variable *x*, *n* corresponds to the number of points in the recorded data set, and *p*(*x*) resembles the probability distribution function. The probability distribution function was fitted in a Gaussian-function, which enabled to estimate the probability distribution function diameter. The standard uncertainty was obtained using the Gaussian fitting function. The number of pixels contributing to the cross-correlation-peak was normalized for consistency of the recorded data in terms of pixels. The bias error was estimated to be about 0.5 pixels because of the complexity of the evaluation of small peaks in terms of sizing in the probability distribution function. The standard uncertainty was evaluated as 3%.

Dust was gathered from photovoltaic surfaces using soft brushes and kept in sampling bottles. They were characterized by utilizing scanning electron microscopy and energy dispersive spectroscopy (JEOL 6460), and X-ray diffraction (Bruker D8). The dust particles size distribution was evaluated using the particle size analyzer (Malvern Panalytical, Mastersizer 3000), which enabled to classify the size of the dust particles within 10–3.5 mm range through incorporating both red and blue light wavelengths.

## Mathematical analysis

The mathematical analysis covers the film vibration under the sonic excitation and the repelled dust particle dynamics resulted from the film vibration.

### Film vibration under sonic excitations

**Figure 2:**
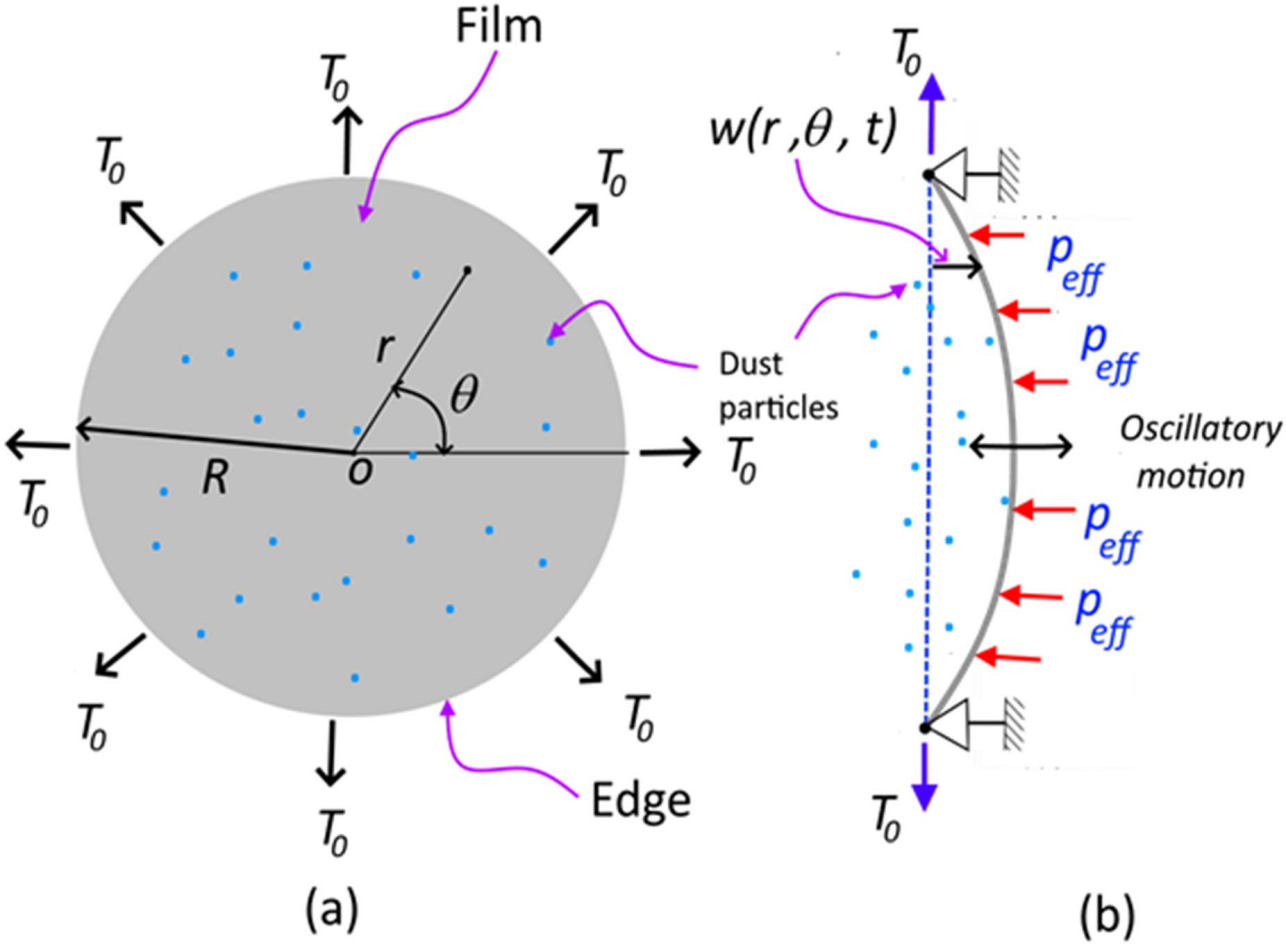
A schematic view of film: (**a**) coordinate system, and (**b**) side view of film and boundary conditions. *T*_*o*_ is in-plane (radial) tensile force, *P*_*eff*_ is the effective pressure created by sonic excitation, and transverse deflection of film.

The circular polyvinyl chloride film is located above the sonic loudspeaker with a standoff distance (spacing between the loudspeaker and the film, Fig. 1) and sonic excitation of the film gives rise to vibrational motion of the film depending on the sonic excitation frequency and the amplitude. The film has a considerably low thickness (14 µm) and it possesses negligible flexural stiffness. The transverse deflection of the film because of the natural frequency of vibration can be formulated from the wave equation. It is worth to mention that the film has a circular shape and it is subjected to a nearly uniform radial force per unit length of in-plane (radial) tensile force (${T}_{0}$) at the film edges. Hence using the cylindrical coordinate system in line with Fig. 2, the equation governing the transverse deflection yields:

1 $$\frac{{\partial }^{2}{w}_{n}(r,\theta , t)}{\partial {t}^{2}}={c}^{2}\left(\frac{{\partial }^{2}{w}_{n}(r,\theta , t)}{\partial {r}^{2}}+\frac{1}{r}\cdot \frac{\partial {w}_{n}(r,\theta , t)}{\partial r}+\frac{1}{{r}^{2}}\cdot \frac{{\partial }^{2}{w}_{n}(r,\theta , t)}{\partial {\theta }^{2}}\right)$$

Here, $c=\sqrt{\frac{{T}_{0}}{\rho h}}$ is the speed of propagation of the transverse wave on the film surface, $\rho$ is the film density, $t$ is time, $h$ is film thickness, ${w}_{n}(r,\theta , t)$ is the transverse deflection. The boundary conditions should satisfy that, at the edges ($r=R$), ${w}_{n}(R,\theta ,t)=0$, i.e. zero transverse displacements at the film edges. In addition, initially, the film is considered to be at rest, i.e. ${w}_{n}(r,\theta ,0)=0$ and as the time approaches infinity time derivative of transverse displacement approaches to zero, i.e. $\frac{\partial {w}_{n}(r,\theta , t)}{\partial t}=0$. The mathematical arrangement of the solution of Eq. (1) is provided in the Supplement (S1). The solution yields:

2 $${w}_{n}\left(r,\theta , t\right)=\left({A}_{1}\cdot \mathrm{cos}\left(c\lambda t\right)+{B}_{1}\cdot \mathrm{cos}\left(c\lambda t\right)\right)\cdot {J}_{m}\left({\lambda }_{mn}r\right)\cdot \left({A}_{2}\mathrm{cos}\left(m\theta \right)+{B}_{2}\mathrm{sin}(m\theta )\right)$$

Here, ${A}_{1}$, ${B}_{1}$, ${A}_{2}$ and ${B}_{2}$ are constants, $\omega =c\lambda$ is the angular frequency, $m=\mathrm{0,1},2,\ldots$. are constants that represent the number of diametral lines with zero deflection. ${J}_{m}$ is the Bessel functions of zero order. The natural frequency of the vibrating film is:

3 $${f}_{mn}=c{\lambda }_{mn}=\frac{c\cdot {k}_{mn}}{2\pi R}=\frac{{k}_{mn}}{2\pi R}\sqrt{\frac{{T}_{0}}{\rho h}}$$

Here: ${k}_{mn}$ can be obtained from the Bessel function of the first kind, $m$ is an integer that represents the number of circumferential lines with zero deflection and $n$ is an integer that represents the number of diametral lines with zero deflection. Hence, $m$ and $n$ can be used to characterize the modes' shape. It is worth to mention that: $R\left(r\right)={J}_{m}\left({\lambda }_{mn}r\right)$ for $m=\mathrm{0,1},\ldots, n=\mathrm{1,2},\ldots$ and ${\lambda }_{mn}=\frac{{k}_{mn}}{R}$ and ${k}_{mn}$ is the n-th positive root of ${J}_{m}$.

The mode shapes of the vibrating film are also modeled numerically via solving Eq. (1) with the boundary conditions defined at $r=R$ then ${w}_{n}(R,\theta ,t)=0$. The Ordinary Differential Equation solver of the COMSOL Multiphysics finite element code is used to obtain the numerical solution. Since the vibrating film has a small thickness (low dimension as compared to its radius), it is represented as a 2D elastic film having properties given in Table 1. In the numerical solution, a uniform radial tensile force per unit length of 1.12 is applied at the edges of the film and the transverse deflection is constrained at the edges. The film is meshed with 5418 triangular elements and the grid independence tests demonstrate that the number of elements leads to the converged solution of Eigenvalues.

**Table 1:** Parameters used to compute film mode shapes.

Parameter	Value
Film radius, $R (\mathrm{mm})$	60
Film density, $\rho (\mathrm{kg}/{\mathrm{m}}^{3})$	930
Film thickness, $h (\mathrm{mm})$	14 × 10^−3^
In-plane (radial) tensile force, ${T}_{0} (\mathrm{N}/\mathrm{m})$	1.12
Elastic modulus, $E (\mathrm{MPa})$	880
Poisson’s ratio, $v$	0.38

Since the film is excited by the sound waves via a loudspeaker, the forced vibrational analysis of the film needs to be considered. Hence, after obtaining the mode shapes of the film, a pulsating (sinusoidal) pressure is applied at the film bottom surface resembling the sound waves emanating from the loudspeaker. Generally, sound waves are considered to be pressure waves resulting from the change in pressure from that of ambient. The propagating sine wave can be represented as:

4 $$\Delta P={\Delta P}_{max}\mathrm{sin}(\omega t-kx)$$

Here, $\Delta P=P-{P}_{atm}$ is the change in pressure, ${\Delta P}_{max}$ is the amplitude (or maximum) pressure change, $k=\frac{2\pi }{\lambda }$ is the wavenumber, $\omega =\frac{2\pi }{T}=2\pi f$ is the angular frequency, $x$ is spatial coordinate, $t$ is time. Hence, the transverse deflection of the film under the forced vibration, ${w}_{f}(r,\theta , t)$, can be expressed as:

5 $$\rho h\frac{{\partial }^{2}{w}_{f}(r,\theta , t)}{\partial {t}^{2}}={T}_{0}\left(\frac{{\partial }^{2}{w}_{f}(r,\theta , t)}{\partial {r}^{2}}+\frac{1}{r}\cdot \frac{\partial {w}_{f}(r,\theta , t)}{\partial r}+\frac{1}{{r}^{2}}\cdot \frac{{\partial }^{2}{w}_{f}(r,\theta , t)}{\partial {\theta }^{2}}\right)+\Delta {P}_{max}\mathrm{sin}(2\pi ft)$$

Since the experiment is carried out at low-frequency ranges, the vibrational mode (0,1) dominates and the solution to ${w}_{f}\left(r,\theta ,t\right)$ can be considered to be axisymmetric. Hence, Eq. (5) becomes a function of $r$ and $\theta$, i.e.:

6 $$\rho h\frac{{\partial }^{2}{w}_{f}(r, t)}{\partial {t}^{2}}={T}_{0}\left(\frac{{\partial }^{2}{w}_{f}(r, t)}{\partial {r}^{2}}+\frac{1}{r}\cdot \frac{\partial {w}_{f}(r,t)}{\partial r}+\frac{1}{{r}^{2}}\cdot \frac{{\partial }^{2}{w}_{f}(r, t)}{\partial {\theta }^{2}}\right)+\Delta {P}_{max}\mathrm{sin}(2\pi ft)$$

The conditions to be satisfied for the solution of Eq. (6) are: ${w}_{f}\left(R,0\right)=0$ and $\frac{\partial w\left(R,0\right)}{\partial t}=0$ at $t=0$.

The exact solution of Eq. (6) can be expressed as^28^:

7 $${w}_{f}\left(r,t\right)=\frac{\Delta {P}_{max}{c}^{2} }{{\omega }^{2}{T}_{0}}\mathrm{sin}\left(\omega t\right)\left(\frac{{J}_{0}\left(\frac{\omega r}{c}\right)}{{J}_{0}\left(\frac{\omega R}{c}\right)}-1\right)-\frac{2\Delta {P}_{max}\omega c}{\alpha {T}_{0}}\sum_{s=1}^{\infty }\frac{\mathrm{sin}\left(c{\alpha }_{s}t\right){J}_{0}(r{\alpha }_{s})}{{\alpha }_{s}^{2}({\omega }^{2}-{c}^{2}{\alpha }_{s}^{2}){J}_{0}{^{\prime}}(r{\alpha }_{s})}$$

Here, $s=\mathrm{1,2},\ldots$, ${\alpha }_{s}$ are the roots of ${J}_{0}\left(az\right)=0$, ${I}_{0}\left(z\right)=1+\frac{{z}^{2}}{{2}^{2}}+\cdots$, ${I}_{0}\left(iz\right)={J}_{0}(z)$, and ${I}_{0}^{{\prime}}\left(iz\right)=-i{J}_{0}{^{\prime}}(z)$.

Because of the consideration of consistency of numerical vibrational mode assessment of the film, the film displacement is also predicted numerically by solving Eq. (6) incorporating the conditions. The COMSOL Multiphysics Differential Equation Solver is used to solve Eq. (6). The vibrating film has a low thickness and it is represented as a 2D elastic film with properties given in Table 1. In addition, a sinusoidally varying force (source term), $\Delta {P}_{max}\mathrm{sin}(2\pi ft)$ is introduced from the film bottom surface. The transverse deflection is constrained at the edges and the film is meshed with 5418 triangular elements. The grid independence tests are carried out securing the grid-independent results.

### Dynamic analysis of repelling dust particles

**Figure 3:**
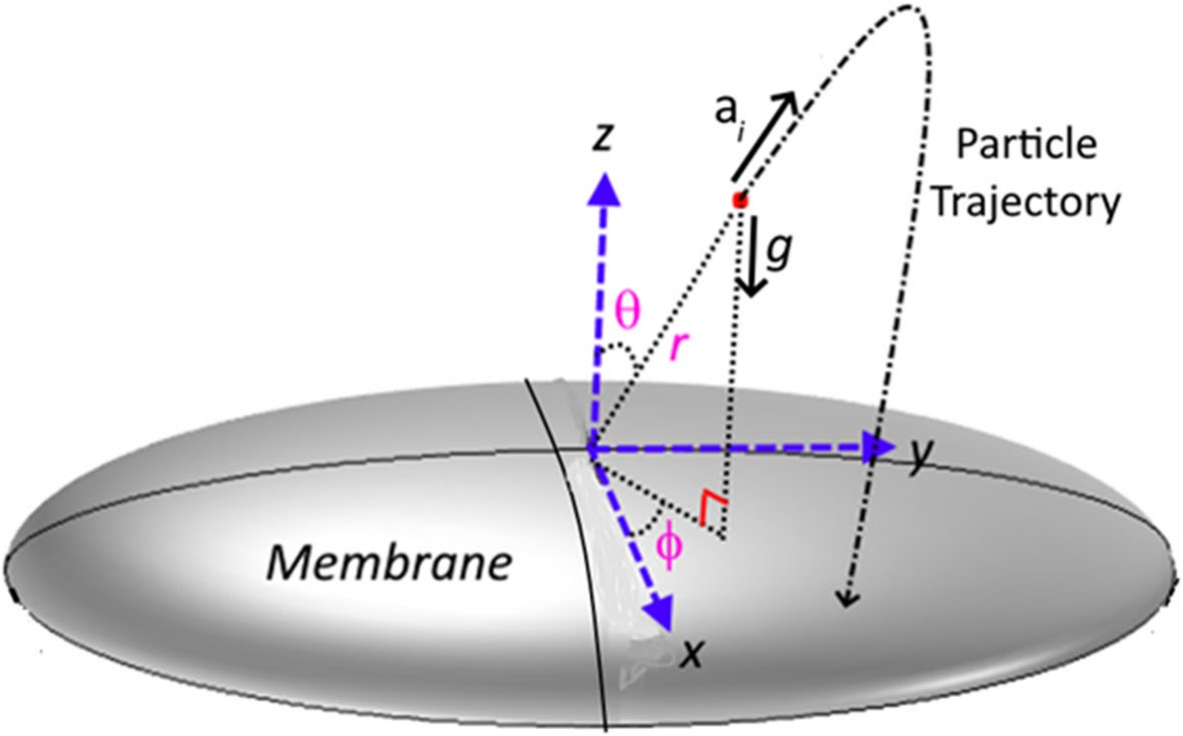
Schematic view of polyvinyl chloride film surface, coordinate system and dust particle trajectory.

The dust particles are repelled from the film surface under the influence of film acceleration because of the sonic excitations. The inflight particle (repelled dust particles from the film surface) motion can be presented in the spherical coordinate system ($r,\theta ,\phi , t$). Figure 3 shows schematically the film and inflight particle in the coordinate system. The formulation of the forces acting on the inflight particle is given in the Supplement (S2). Hence, the forces acting on the inflight dust particle at the onset of repelling from the film surface are:

8 $$\sum {F}_{r}=m\left(\frac{{d}^{2}r}{d{t}^{2}}-r{\left(\frac{d\phi }{dt}\right)}^{2}{\mathrm{sin}}^{2}\upphi -r{\left(\frac{d\uptheta }{dt}\right)}^{2}\right)=-mgcos\theta -D\frac{dr}{dt}+{F}_{ac}$$

9 $$\sum {F}_{\theta }=m\left(2\frac{dr}{dt}\cdot \frac{d\theta }{dt}+r\frac{{d}^{2}\theta }{d{t}^{2}}-r\frac{{d}^{2}\phi }{d{t}^{2}}cos\theta \cdot sin\theta \right)=-mgsin\theta cos\phi -D\frac{d\theta }{dt}$$

10 $$\sum {F}_{\phi }=m\left(2\frac{dr}{dt}\cdot \frac{d\phi }{dt}{\sin\theta }+\mathrm{r}\frac{{d}^{2}\phi }{d{t}^{2}}{\sin\theta }+2\mathrm{r}\frac{d\phi }{dt}\cdot \frac{d\uptheta }{dt}cos\theta \right)=-mgsin\theta sin\phi -D\frac{d\phi }{dt}$$

Here, ${F}_{i}$ is the inertia force, $m$ is the particle mass, $D$ is the drag force term according to Stokes’ hypothesis, ${d}_{p}$ is the particle diameter, and ${F}_{ac}$ is the acoustic force term. Moreover, the airflow around the dust particle occurs at low Reynold number $(Re)$ and the Stokes’ hypothesis can be adopted formulating the drag force, i.e.: $D=3\pi \mu {d}_{p}$. In addition, the acoustic radiation force on a particle moving in a viscous fluid is formulated previously^29^ and this formulation is used. The acoustic force yields ${F}_{ac}=\pi \frac{{d}_{p}^{3}}{8}kf{E}_{ac}$, here $k$ is the wave number, ${E}_{ac}=\frac{1}{2{\rho }_{0}{c}_{0}^{2}}{p}_{rms}^{2}$ is acoustic energy density and $k$ is the dipole scattering coefficient. The dipole scattering coefficient is taken as: $f=\frac{6{\left(1-\rho \right)}^{2}\left(1+\delta \right)\delta }{{\left(1+2\rho \right)}^{2}+9\left(1+2\rho \right)\delta +\frac{81}{2\left({\delta }^{2}+{\delta }^{3}+\frac{{\delta }^{4}}{2}\right)}}$, where, $\rho =\frac{{\rho }_{p}}{{\rho }_{a}}$ is the particle-to-air density ratio and $\delta =\frac{{\delta }_{b}}{{r}_{p}}$ is the ratio of boundary layer and particle radius. The boundary layer developed on a spherical particle can be obtained from ${\delta }_{b}=\frac{4.53{d}_{p}}{\sqrt{Re}}$^29^. The force term in Eqs. (8–10) can be formulated in terms of particle mass and acceleration, i.e.: ${m}_{p}\frac{{d}^{2}r}{d{t}^{2}}$, ${m}_{p}\frac{{d}^{2}\theta }{d{t}^{2}}$, ${m}_{p}\frac{{d}^{2}\phi }{d{t}^{2}}$, where *m*_*p*_ is the particle mass. In consistency with the solutions for the film vibrational motion, the numerical solution of Eqs. (8–10) can obtained using the Differential Equation solver of COMSOL Multiphysics finite element code. It is worth to mention that the second order Euler backward difference scheme is used to discretize the equations and the nonlinear solution is obtained with the aid of the Newton-Rapson method. Since the accuracy of the predictions is limited by the selection of the time increment, the time step is set at 10^−8^ s in the numerical simulations. Table 2 gives the parameters used in the simulations.

**Table 2:** Parameters used in the particle dynamic analysis.

Parameter	Value
Density of air, $\rho \left(\frac{\mathrm{kg}}{{\mathrm{m}}^{3}}\right)$	1.2
Density of particle, ${\rho }_{p} \left(\frac{\mathrm{kg}}{{\mathrm{m}}^{3}}\right)$	2800
Large Particle diameter, ${d}_{p}$ (mm)	0.1732
Initial radial position, ${r}_{0}$ (mm)	200
Initial rotation angle, ${\theta }_{0}$	5°
Initial tilt angle, ${\phi }_{0}$	5°
Adhesion force, ${F}_{ad}$	2 × 10^−12^ N

## Results and discussion

Environmental dust characteristics and dust particles mitigation from transparent polyvinyl chloride film are examined. The mechanisms of the dust particles repelling via sonic excitation are explored and the dynamics of the repelled particles are formulated. The findings of the repelled particle heights are compared with their counterparts obtained from the experiments. The optical transmittance of the dust mitigated surfaces is also evaluated.

### Dust and surface characteristics

**Figure 4:**
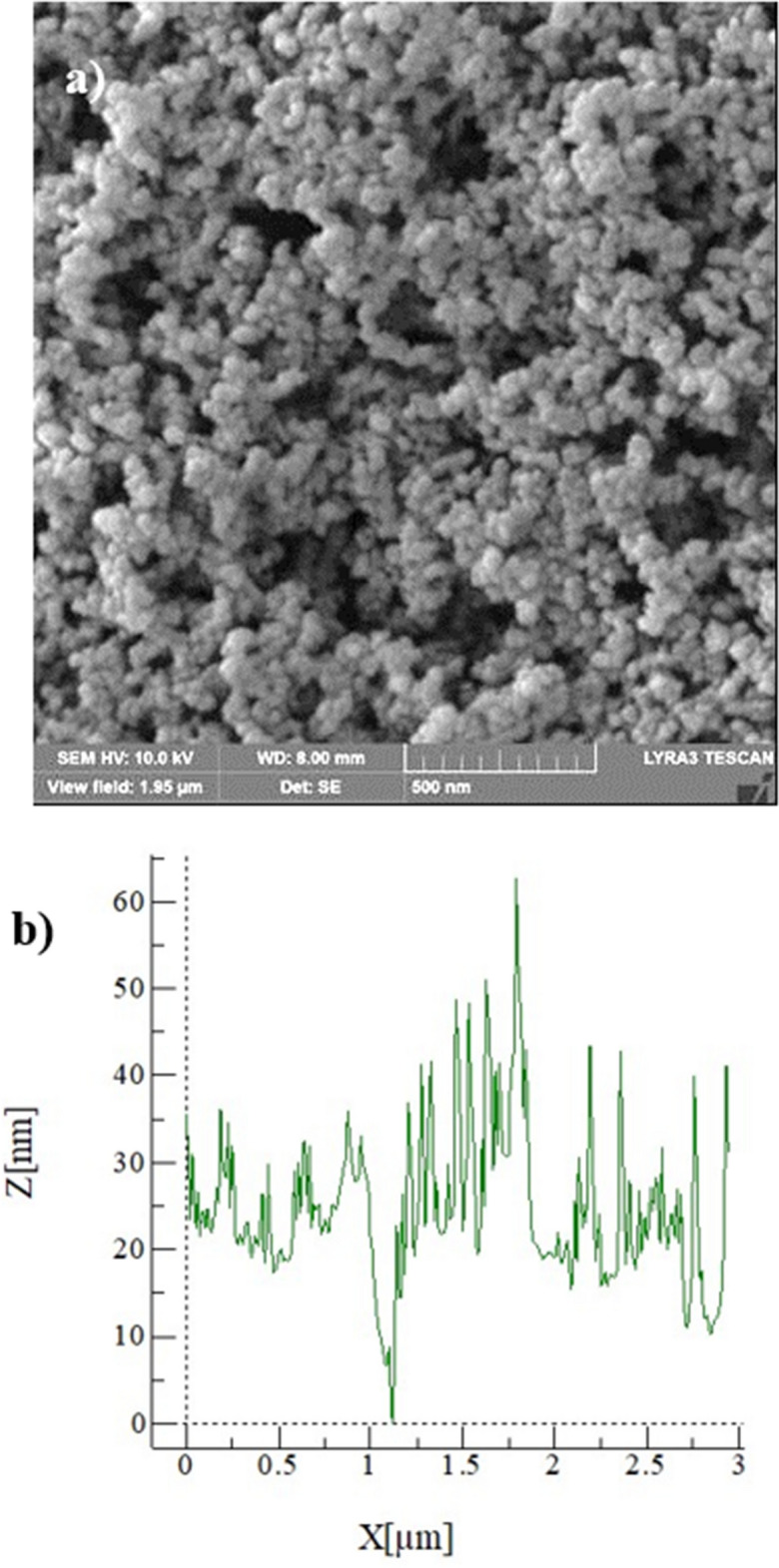
SEM image of coating surface and line scan: (**a**) SEM micrograph, and (**b**) AFM line scan of coating surface.

**Figure 5:**
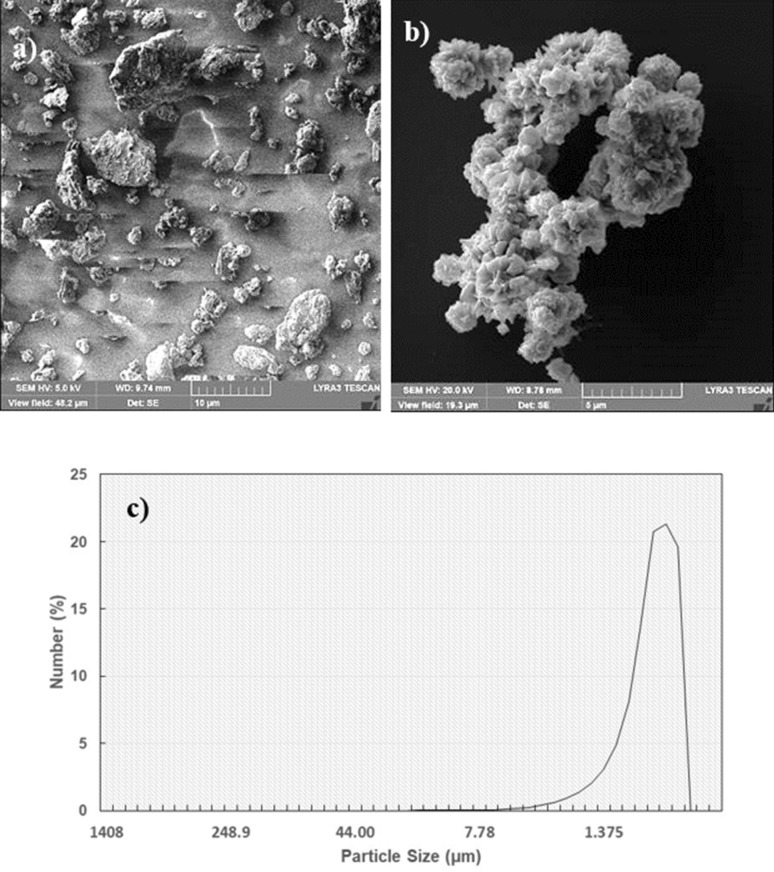
SEM micrograph of dust particles: (**a**) various size and shapes dust particles, (**b**) clustered small size dust particles, and (**c**) size distribution of dust particles.

**Figure 6:**
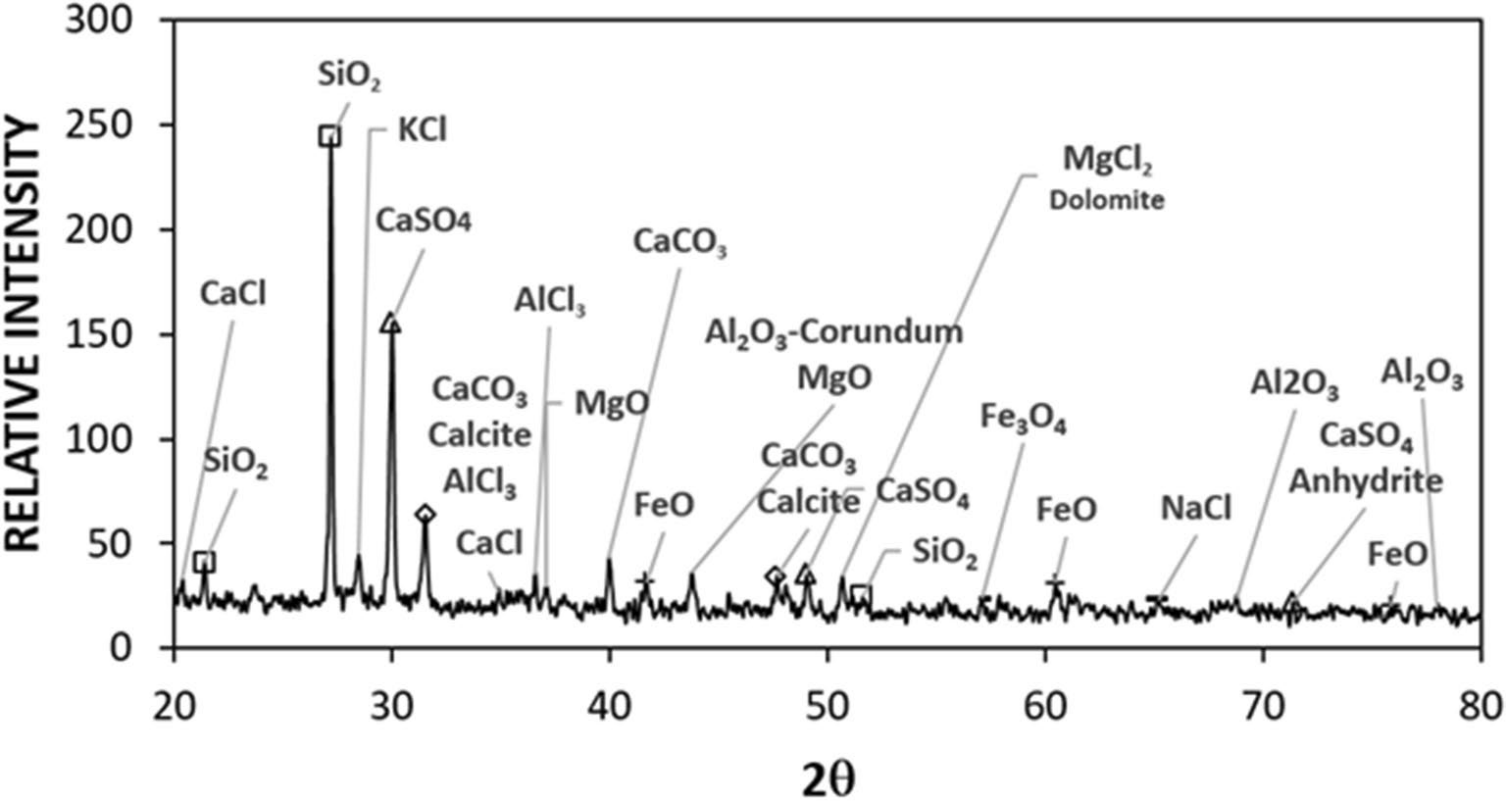
X-ray diffractogram of dust.

Figure 4a shows SEM microimages and Fig. 4b depicts the AFM line scan of the hydrophobized film surface. The silica particles have sizes almost 30 nm (Fig. 4a) and form a clustered layer on the sample surface. Small texture height is noted along the line scan (Fig. 4b) and the average roughness is about 40 nm. The peaks and valleys in the texture of the surface (Fig. 4b) demonstrates that silica particles clustered while forming small peaks on the surface, which creates a Lotus effect on the liquid droplets while reducing droplet contact angle hysteresis. The coated surface wetting is measured and the contact angle of the coated surface is about 150° ± 2° and hysteresis is 4° ± 2°, i.e. coating surface demonstrates superhydrophobic identity. On the other hand, dust is collected from PV panel surfaces in Dhahran, Saudi Arabia, and characterization tools are used to evaluate particle size, shapes, and elemental constitutes. Figure 5a,b show SEM microimages of dust particles while Fig. 5c shows dust particle distribution. Dust have different shapes and sizes (Fig. 5a). In addition, particularly, small particles agglomerate forming the clusters (Fig. 5b). The agglomeration and adherence of small particles are because of ionic forces formed on these particles. Elemental constitutes obtained from EDS analysis demonstrate that dust retains several elements, such as Si, Na, Ca, K, S, Fe, Cl, and O (Table 3). The quantification of elemental constitutes does satisfy the stoichiometric ratio for small particles (2 µm ≤) particularly alkaline salt components (NaCl and KCl) as observed in Table 3, which is also reported in the earlier work^17^. This contributes to dust agglomeration (Fig. 5b). Dust shapes can be evaluated based on the shape factor (${A}_{sh}= \frac{{P}^{2}}{4\pi A}$ , where *P* is perimeter of the dust particle and *A* is area of dust cross-section) and aspect ratio (${A}_{s}=\frac{\pi {({L}_{l})}^{2}}{4A}$, here *L*_*l*_ is the largest projection length)^30^. The microscopic methods are utilized to determine the feature of dust geometry. The shape factor, mostly, ranges from 0.5 to 3. and one corresponds to dust with the almost circular feature. There is no clear distribution of shape factor with a range of dust sizes of 2–5 µm. As dust becomes greater than 5 µm, the shape factor becomes almost 3. However, it becomes almost one for small size dust (≤ 0.8 µm). The aspect ratio varies considerably with dust sizes and aspect ratio approaches almost one for small size dusts ((≤ 0.8 µm). In addition, a simple mathematical expression correlating the shape factor and the aspect ratio in terms of dust sizes could not obtained. The particle size of the dust varies in micrometer to nanometer ranges with an average of 1.2 µm (Fig. 5c). Figure 6 demonstrates the X-ray diffraction data for dust. The salt compounds (NaCl and KCl), calcite, silica peaks are apparent. The peaks of iron and silicon overlap and sulfur peak could be anhydrite or gypsum (CaSO_4_) while iron (Fe) peak corresponds to clay-aggregated hematite (Fe_2_O_3_). Moreover, dust adhesion on coated sample surfaces is calculated adopting the technique developed earlier^31^. The AFM probe deflection in friction mode is utilized determining the dust particle adhesion on the sample surface. Hence, $F=k{\sigma }_{d}\Delta V$, here *k* is spring constant (N/m) of probe tip, *σ*_*d*_ is slope of probe deflection (*Δz*/*ΔV*, m/V), and *ΔV* is probe output recorded (mV) onset of deflection. The AFM probe used has the characteristics of $k{\sigma }_{d}$ = 5.80275 × 10^−15^ N/mV. The particle of about 0.9 µm size on the hydrophilic sample surface (as received), the output probe voltage is recorded as 380 mV and equation ($F=k{\sigma }_{d}\Delta V$) yields the adhesion force of 2.21 × 10^−12^ N. Similarly, for about 8 µm size particle, the probe output is 320 mV, which yields the adhesion force of 1.86 × 10^−12^ N. Hence, the adhesion force increases almost 16% as the particle size reduces from 8 to 0.9 µm, which demonstrates the increased adhesion of small particles on the sample surface. The adhesion force calculations are repeated for the hydrophobized sample surfaces. The probe output reading for 0.9 µm size particle on the hydrophobized (coated) sample surface is 205 mV, which gives the adhesion force of 1.45 × 10^−12^ N, and for 10 µm size particle on coated sample surface yields the probe output of 170 mV, i.e. the adhesion force for 10 µm size particle is 0.98 × 10^−12^ N. Therefore, hydrophobizing the sample surface reduces the particle adhesion force by almost 35% for 0.9 µm particle and about 47% for 8 µm particle. Consequently, hydrophobizing the surface becomes more effective towards reducing the particle adhesion on the surfaces, particularly for large size particles. Adhesion force experiments are repeated 10 times to secure accurate data. The standard deviation of the data points due to repeatability ($s=\sqrt{\frac{1}{N-1}\sum_{i=1}^{N}{({x}_{i}-\stackrel{-}{x)}}^{2}}$, where *N* is the number of repeats, *x*_i_ is the output recorded (mV), $\stackrel{-}{x}$ is the mean value of probe output) is estimated at about 7%.

**Table 3:** Elemental composition of dust particles (wt%).

	Size	Si	Ca	Na	S	Mg	K	Fe	Cl	O
Collected	≥ 1.2 μm	11.8	8.3	2.2	1.3	2.5	0.8	1.2	0.4	Balance
Collected	< 1.2 μm	10.2	7.3	2.7	2.5	1.3	1.2	1.1	1.1	Balance

### Dynamics of repelling dust particles

**Figure 7:**
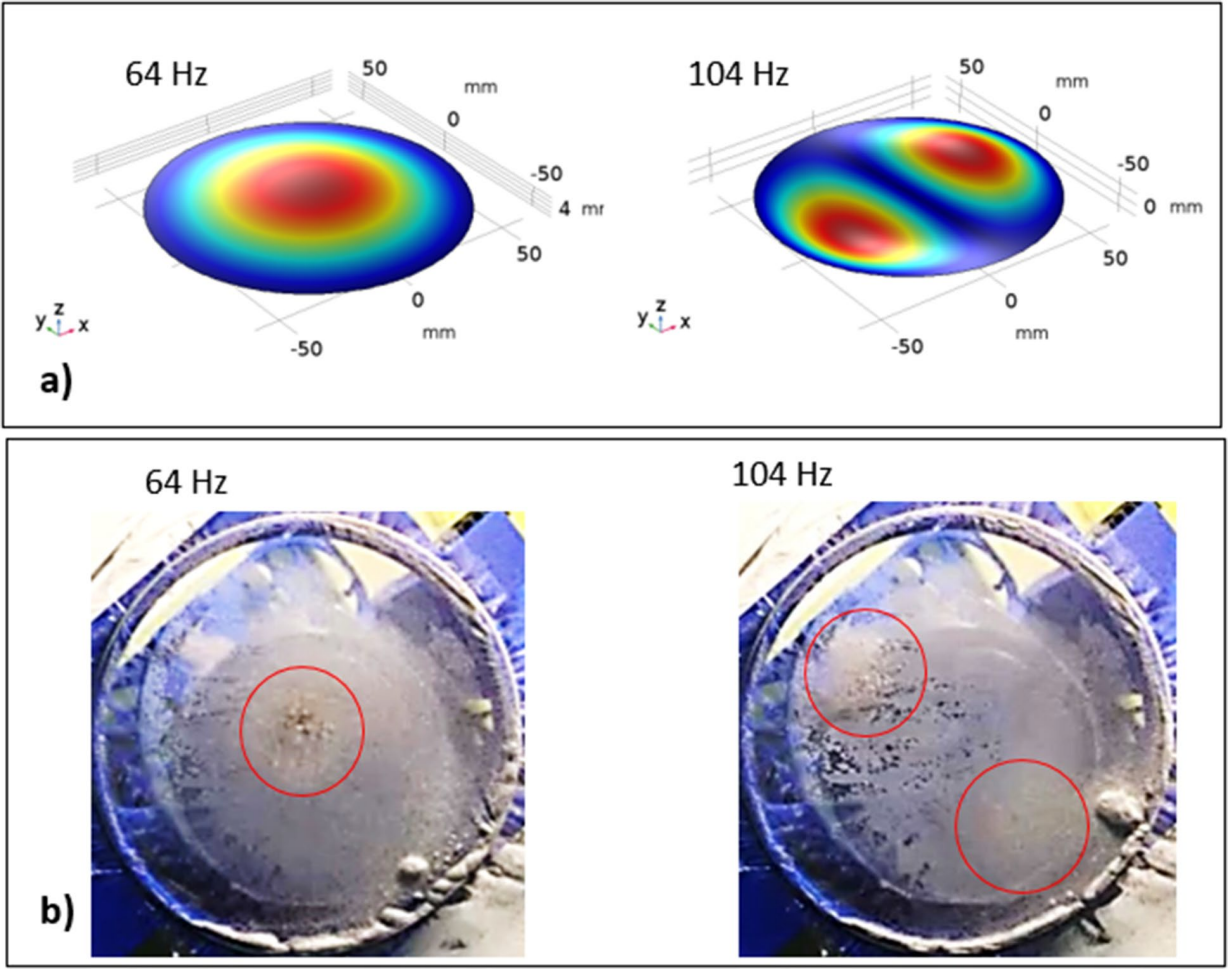
Mode of vibration at two sonic excitation frequencies (64 Hz and 104 Hz): (**a**) predictions, and (**b**) experimental.

**Figure 8:**
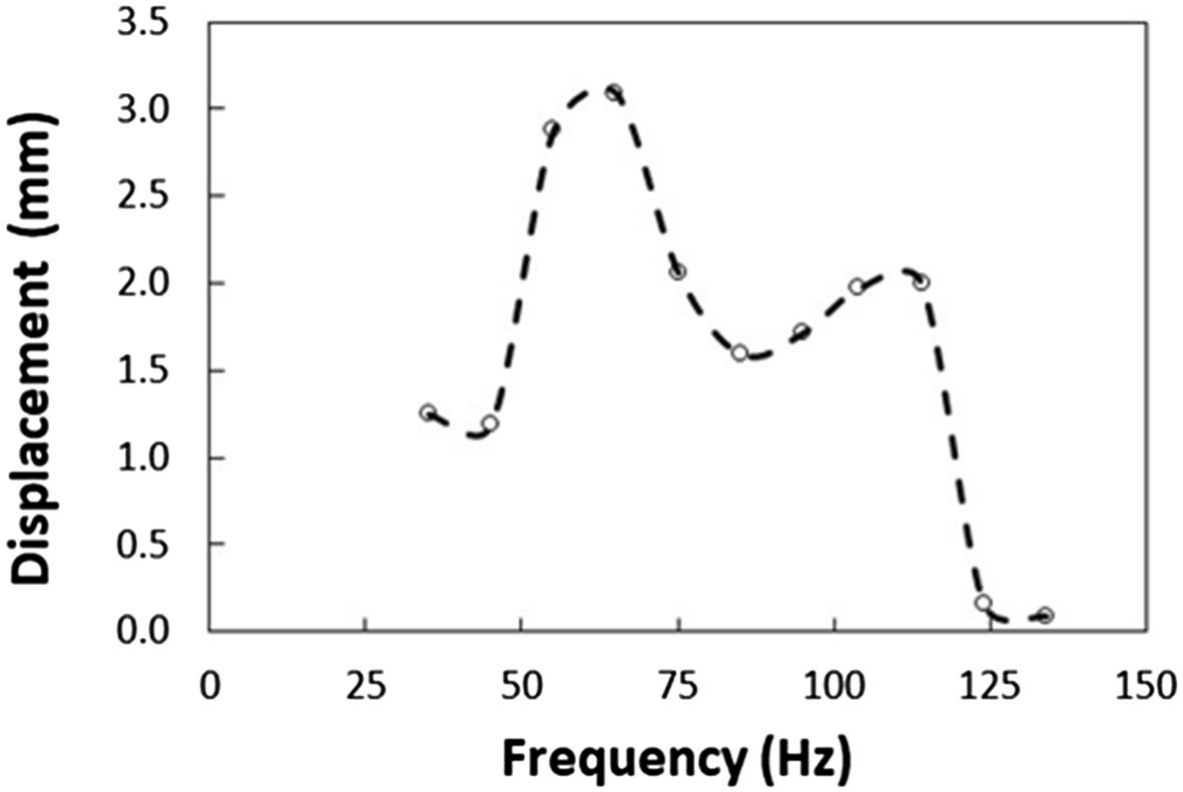
Transverse displacement of film with sonic excitation frequency.

**Figure 9:**
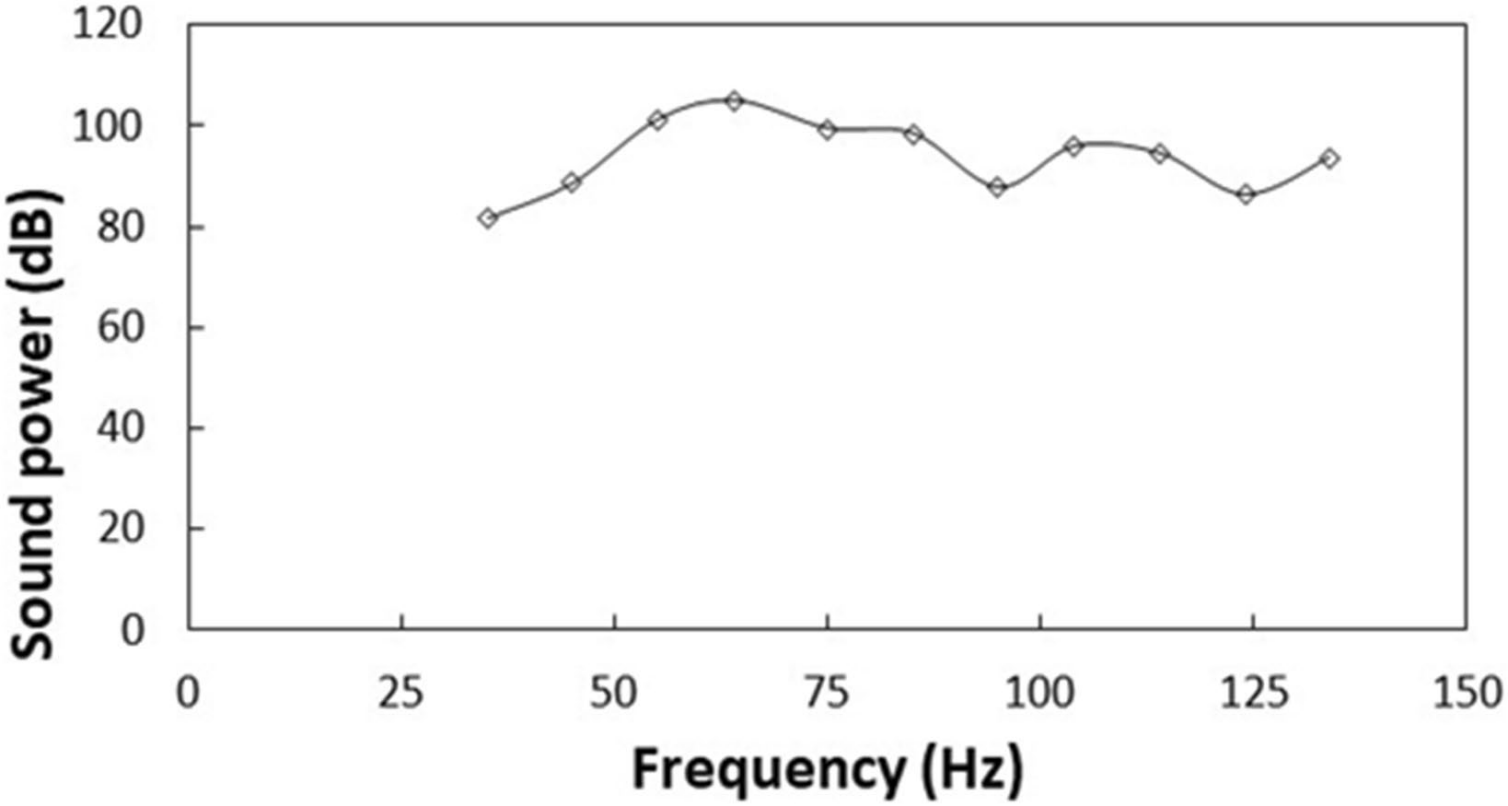
Sound power resulted on film with sonic excitation frequency.

**Figure 10:**
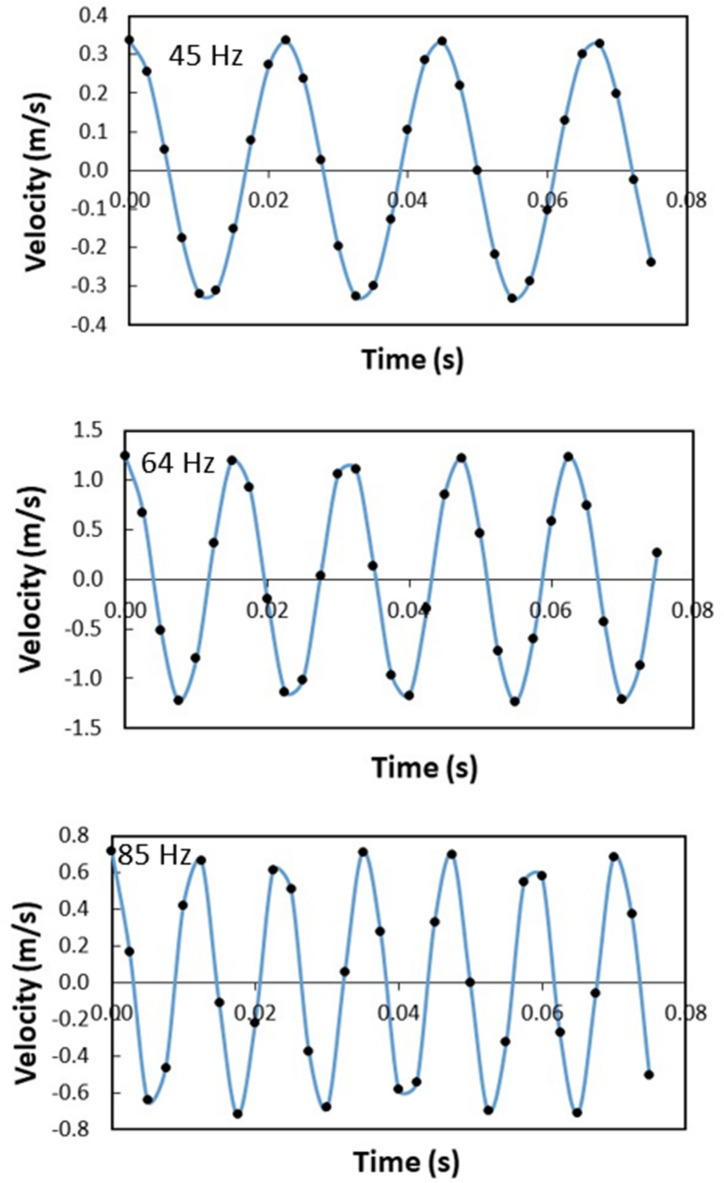
Film velocity with time at different sonic excitation frequencies. The standoff distance is 30 mm.

The polyvinyl chloride (PVC) circular thin film with 14 µm thickness is excited by the sonic waves (sonic radiation) within 35–300 Hz and 80–100 dB amplitude from the bottom of the film while the particles are located on the top surface of the film (Fig. 1). The film possesses very negligible flexural stiffness and the mode of deflection of the film can be obtained from Eqs. (2) and (3). Moreover, initially, the assessment of the mode of film vibration is carried out both numerically (Eqs. 2 and 3) and experimentally. Figure 7a shows the mode of vibration of the film at two different sonic excitation frequencies (64 Hz and 104 Hz) while Fig. 7b depicts the corresponding modes obtained from the experiments. Tables 4 and 5 give the data for the mode of shapes and natural frequencies of film vibration. At low excitation frequencies (45 Hz–85 Hz), single-mode results while at high excitation frequency four-mode results. Since the dust particles have small sizes, the mode of film vibration results in the clustering of the dust particles on the film surface (Fig. 7b). The transverse displacement (along the y-axis) of the film surface is evaluated experimentally using high speed camera data incorporating the tracker program at low frequencies one mode shape (1,0) of the film vibration (Fig. 7b). Figure 8 shows the transverse displacement of the film surface with different excitation sound frequencies and resulting sound power. The displacement remains maximum for the frequency of 64 Hz because of the resonant frequency. To evaluate the effect of the location of the sound excitation reference to the film location (standoff distance), various tests are conducted. Figure 9 shows the sound power variation with the frequency. The local peak sound power occurs at a frequency of 64 Hz. Further tests are carried out to assess the influence of the input voltage of the sound wave generator on the dynamic characteristics of the film. Table 6 gives the data for film dynamic characteristics. In addition, the amplitude of film vibration at various frequencies is also obtained from the analytical (Eq. 7) and numerical (Eq. 6, COMSOL simulations) approach and the values are included in Table 6. The findings reveal that the input voltage of 8.4 V for loudspeakers with *d* = 30 mm standoff distance (Fig. 1) results in the maximum sound power, which is used for dust mitigation study, i.e. the sound power 104.9 dB is resulted (Table 6). Moreover, the velocity of the film in the transverse direction is determined from experiments and the findings are shown in Fig. 10. To evaluate the influence of sonic excitation frequency on the film transverse velocity, three frequencies are included in Fig. 10 for comparison. The velocity of the film in the transverse direction follows the oscillatory motion of the film with the same frequency. The maximum amplitude of the transverse velocity is about 1 m/s, which occurs at 64 Hz of the external excitation frequency. It is worth mentioning that the sound power is the maximum for 64 Hz excitation frequency with a standoff distance of 30 mm (Table 6).

**Table 4:** Possible modes shapes for film vibration. The numbers represent coefficients obtained from the Bessel function.

(m,n)	$n=0$	$n=1$	$n=2$
$m=1$	2.4048	3.8317	5.1356
$m=2$	5.5204	7.0155	8.4172
$m=3$	8.6537	10.1735	11.620

**Table 5:** Predicted natural frequencies of film vibration.

Vibration modes	Natural frequencies		
	Experimental	Analytical	Numerical
(1,0)	64.0 ± 5	63.79	63.79
(1,1)	104.0 ± 5	101.64	101.64
(1,2)	124.0 ± 5	136.23	136.24
(2,2)	–	146.43	146.46

**Table 6:** Film vibration characteristics under sonic excitation.

Case #	Frequency (Hz)	Angular Freq. (rad/s)	Distance (mm)	Damping	Amplitude (V)	Mode (m,n)	Measured Sound Power	Pressure amplitude	Film vibrating amplitude (mm)		
							dB	Pa	Experimental	Analytical	Numerical
1	35	219.91	30	No	8.4	–	81.6	0.34	1.249	1.16	0.8
2	45	282.74	30	No	8.4	–	88.6	0.76	1.190	1.1	0.95
3	55	345.58	30	No	8.4	–	101.2	3.25	2.879	2.81	2.75
4	64	402.12	30	No	8.4	(1,0)	104.9	5	3.092	3.01	3.3
5	64	402.12	50	No	8.4	(1,0)	103.8	4.40	2.800	2.71	3
6	64	402.12	70	Yes	8.4	(1,0)	95.6	1.71	1.020	–	–
7	64	402.12	70	No	8.4	(1,0)	101.3	3.30	2.170	2.01	2.3
8	64	402.12	70	No	5.6	(1,0)	99.9	2.80	1.890	1.71	2
9	64	402.12	70	No	2.8	(1,0)	92.9	1.25	0.910	0.75	1.02
10	75	471.24	30	No	8.4	–	99.4	2.65	2.060	–	–
11	85	534.07	30	No	8.4	–	98.4	2.36	1.343	–	–
12	95	596.90	30	No	8.4	–	87.8	0.69	1.714	–	–
13	104	653.45	30	No	8.4	(1,1)	95.9	1.77	1.970	–	–
14	114	716.28	30	No	8.4	–	94.5	1.51	2.000	–	–
15	124	779.11	30	No	8.4	–	86.3	0.58	0.164	–	–
16	134	841.95	30	No	8.4	–	93.7	1.37	0.091	–	–
17	300	1884.96	30	No	8.4	–	92.6	1.21	0.182	–	–

**Figure 11:**
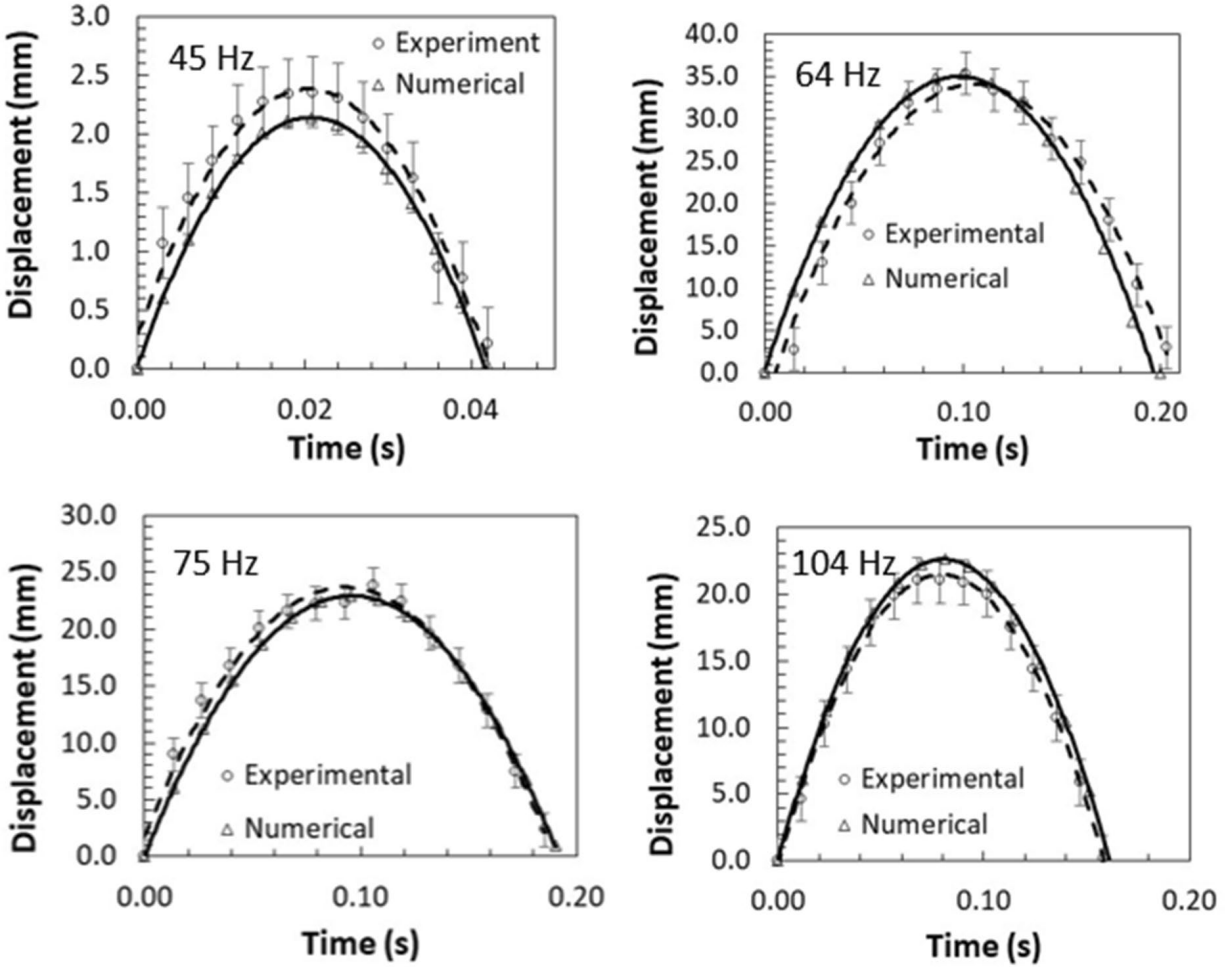
Vertical displacement (height) of inflight dust particle (repelled) predicted from numerical simulations and obtained from experiment. The dust particle initial location on the film surface is (200, 5.74°, 5°).

**Figure 12:**
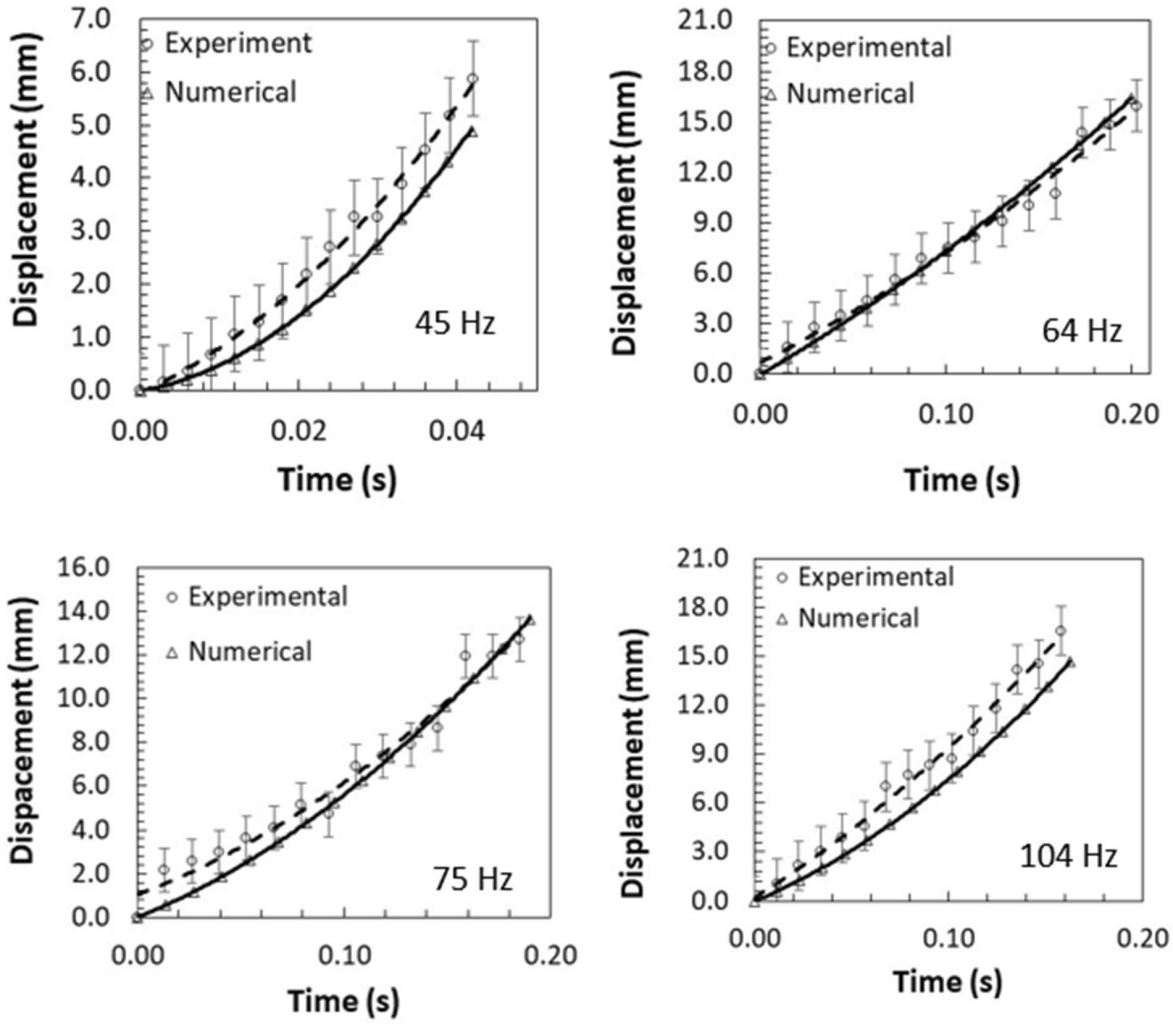
Horizontal displacement (height) of inflight dust particle (repelled) predicted from numerical simulations and obtained from experiment. The dust particle initial location on the film surface is (200, 5.74°, 5°).

**Figure 13:**
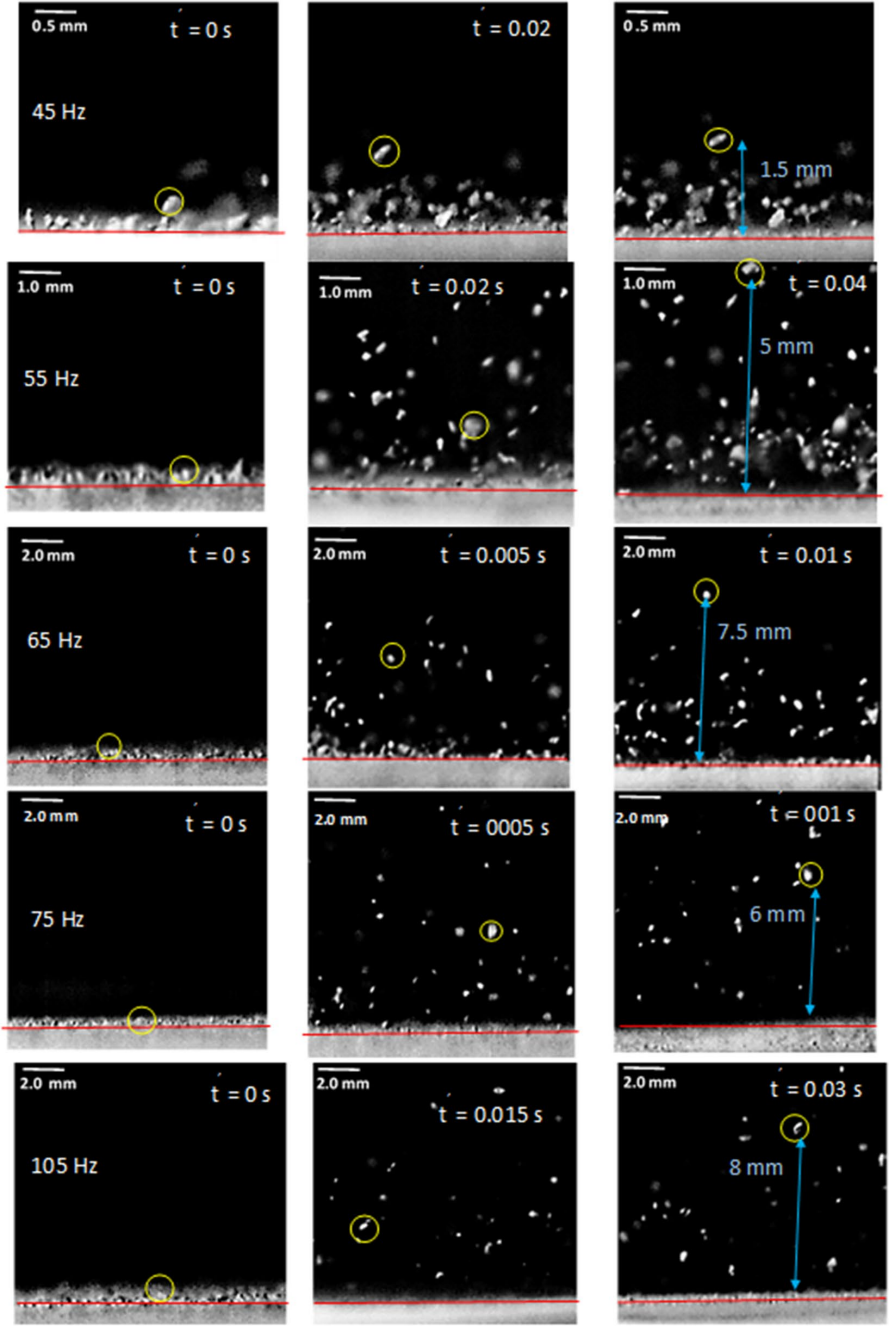

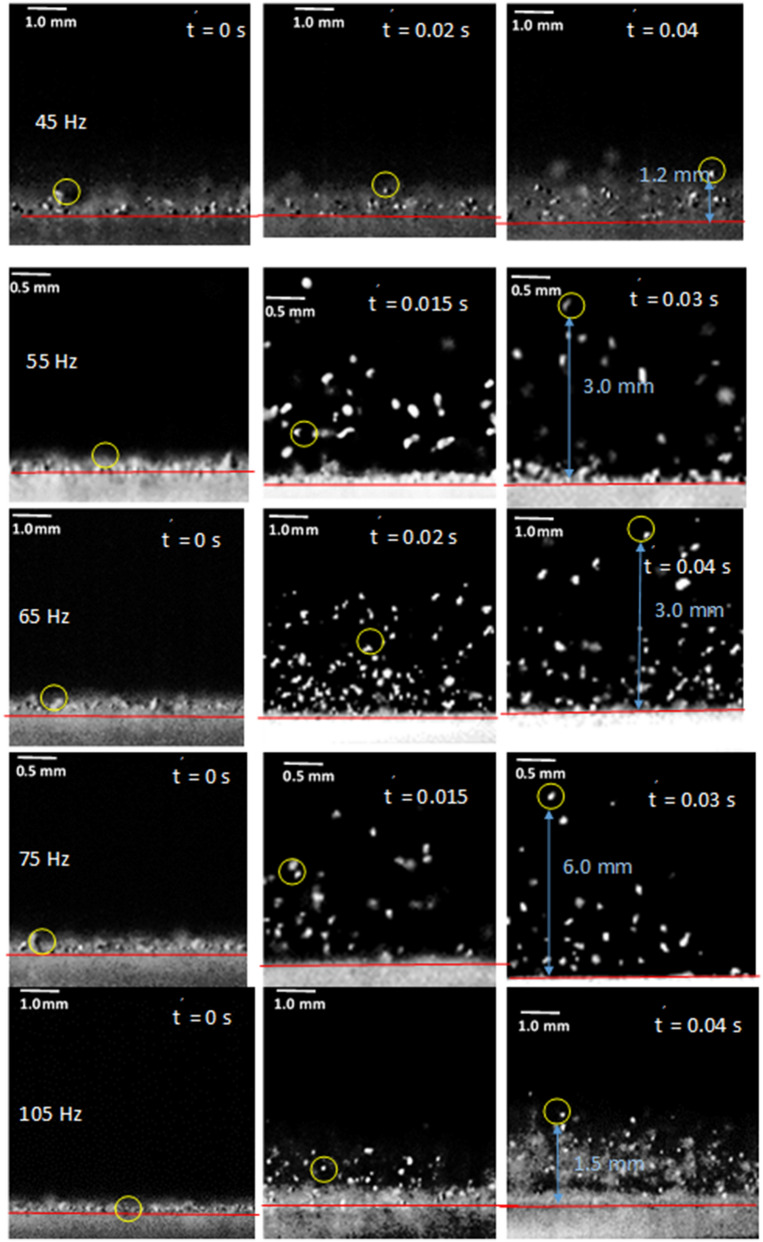
(**a**) Optical images of repelled dust particles on hydrophilic surface at different frequencies and times. (**b**) Optical images of repelled dust particles on hydrophobic surface at different frequencies and times.

**Figure 14:**
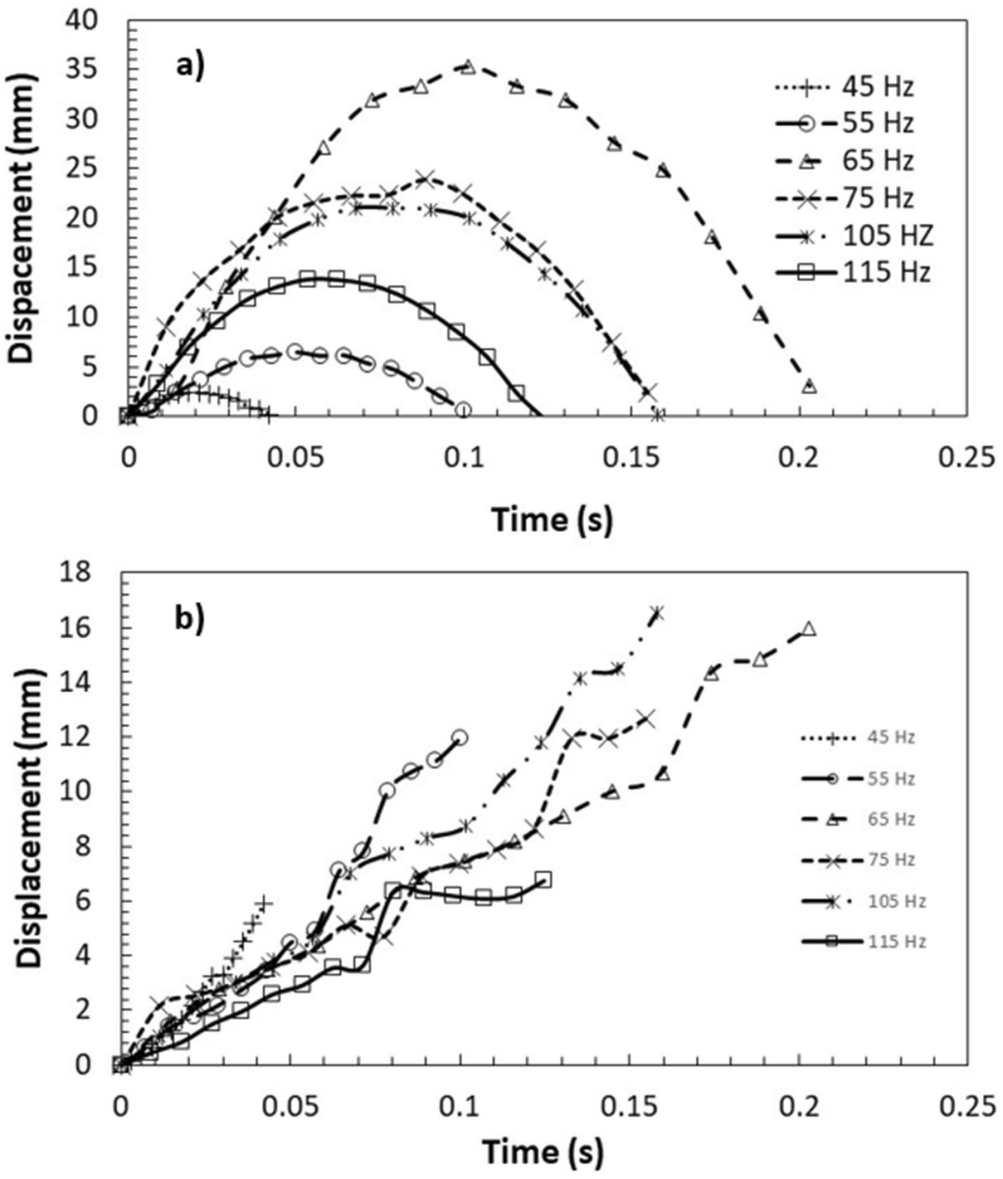
Repelled dust particles vertical and horizontal heights obtained from experiments: (**a**) vertical height, and (**b**) horizontal height. Dust particle initial location on film is (200, 5.74°, 5°).

**Figure 15:**
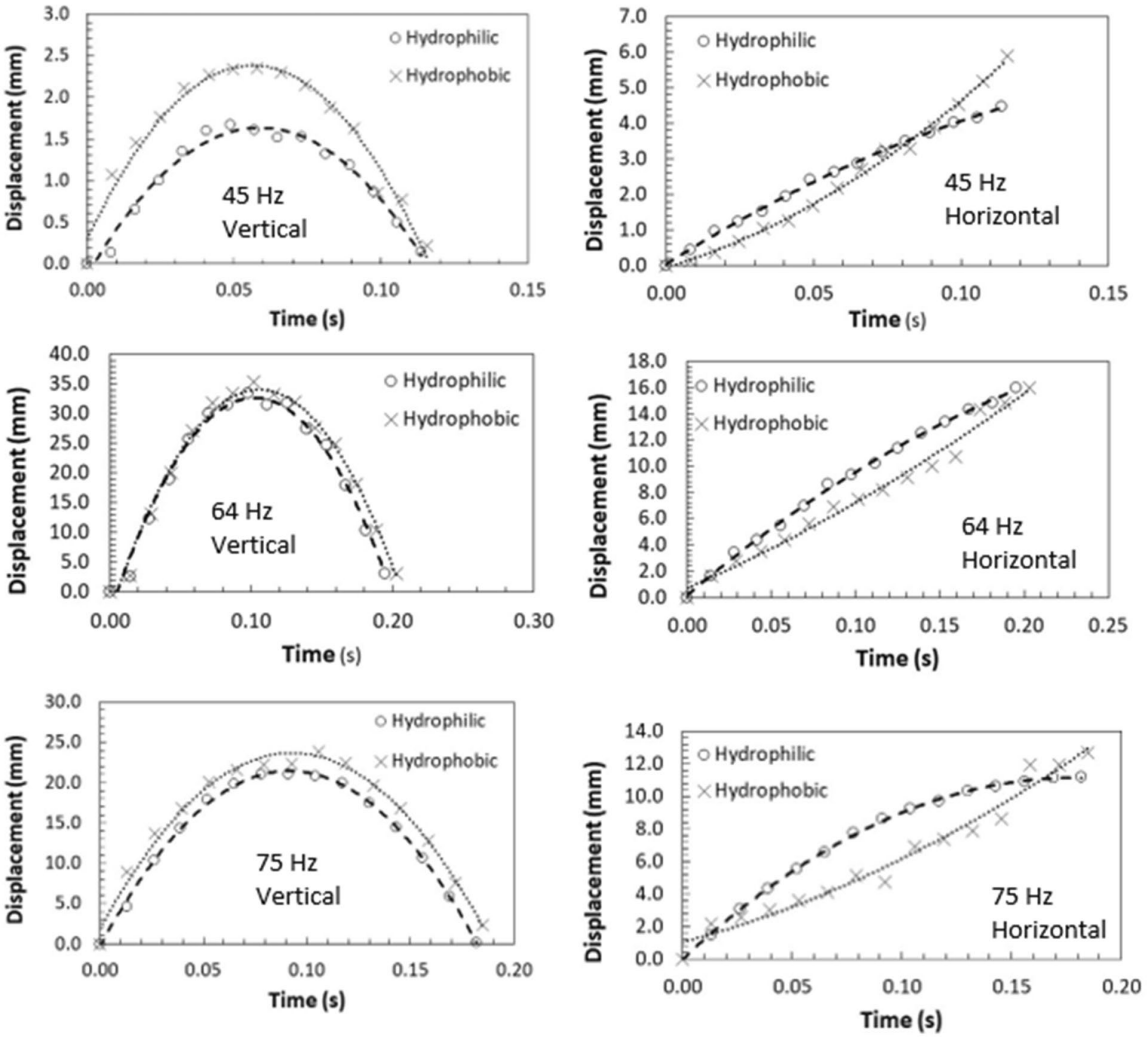
Vertical and horizontal displacement of dust particles on hydrophobic and hydrophilic surfaces with time at different sonic excitation frequencies. Dust particle initial location on film surface is (200, 5.74°, 5°).

Under the sonic excitation, rapid displacement of the film causes dust particle acceleration. The trajectory of the repelled particles are formulated through Eqs. (8–10) and predicted using Differential Equation solver (DE) of COMSOL multi-physics code. Figures 11 and 12 show the temporal behavior of the inflight particle vertical (Fig. 11) and horizontal heights (Fig. 12) obtained from the experimental data using the tracker program (incorporating the high-speed recorded data for hydrophilic surface) and predicted from the numerical simulations, at which the particle is located at (200, 5.74°, 5°) on the film surface for four sonic excitation frequencies (45 Hz, 64 Hz, 75 Hz, and 104 Hz), respectively. Here, the coordinate system ($r,\theta ,\phi$) is according to Fig. 3. It is worth to mention that as the particle acceleration reaches a value that takes over the adhesion and weight of the particle, the particle can depart from the film surface. The vertical height of the inflight dust particle follows a parabolic rise and fall with time. This is because of the force balance between the particle inertia, gravity, and drag forces. The gravitational force over the drag force of the dust particle is in the order of 10^−3^ which demonstrates that the gravitational force has a major influence on the inflight particle deceleration with time. The maximum vertical height of the inflight dust particle occurs at about 0.02 s from its departure for 45 Hz excitation; however, the horizontal location continues to increase with time for a longer duration (0.1 s). Moreover, as the excitation frequency changes, the time occurrence of the maximum vertical displacement differs, i.e. 0.1 s for 64 Hz and 0.08 s for 104 Hz of excitations. This indicates that the inflight time of the repelled particle remains longer in the air for 64 Hz excitation. Hence, the repelled particle trajectory has two-stages such that in the first stage, the particle rises both vertically and horizontally under the influence of the sonic excitation while in the second stage the particle follows a falling trend from its maximum vertical height. This allows the repelled particles landing on the film surface significantly away from their repelling locations on the film, which becomes more apparent for 64 Hz of sonic excitation. This indicates that the particle can be removed from surface of the film while creating the multiple sonic excitations. For inflight particle velocity, the horizontal component of the particle increases while the vertical velocity component becomes zero at the maximum peak location, particularly at 64 Hz excitation frequency. As comparing the experimental findings with the predictions of the inflight particle heights in vertical and horizontal directions, both results are in good agreement. The small differences are because of the experimental errors (7%) and the consideration of uniform density particles, which may change slightly from particle to particle because of varying elemental composition (Table 3). Nevertheless, both findings are in agreement. Figure 13a,b show an optical image of the inflight particles at various times and different sonic excitation frequencies for hydrophilic and hydrophobic film surfaces, respectively. The tracking of a particle at different time steps is marked in Fig. 13a,b. It can be observed that at low (45 Hz) and high (105 Hz) sonic excitation frequencies, the heights of the inflight particles are lower than that of the 64 Hz. In addition, at these frequencies, some particles remain on the film surface, i.e. particle inertia force remains less than the adhesion and weight forces of the particles. This situation can also be seen from Fig. 14a,b, in which the temporal variation of horizontal and vertical heights of the inflight dust particle is shown for various sonic excitation frequencies. In the case of the hydrophobic surfaces, vertical and horizontal heights of the dust particles change. Figure 15 shows the temporal variation of horizontal and vertical heights of the inflight particle at the initial location (200, 5.74°, 5°) on the hydrophilic and hydrophobic film surfaces for different sonic excitation frequencies (45 Hz, 64 Hz, and 75 Hz). It is worth to note that inflight particle heights are obtained experimentally. The inflight particle height on the hydrophobic film becomes larger than its counterpart corresponding to the hydrophilic surface at low excitation frequency 45 Hz. As the excitation frequency increases, the inflight particle height difference becomes small along the vertical line (normal to the film surface); however, some small decrease in the horizontal height of the particle is observed for the hydrophobic surface. Nevertheless, the inflight particle heights in both vertical and horizontal directions become similar to whether the particle is initially located on the hydrophobic or hydrophilic film surfaces as excitation frequency increases to 64 Hz. This is attributed to the inertial force generated on the particle located on the film surface. As the frequency reduces (45 Hz), the film displacement and velocity reduce significantly in vertical and horizontal directions. This lowers the inertial force created on the particle to be repelled from the film surface. Hence, at low excitation frequency, the influence of the particle adhesion force on the particle acceleration becomes critically important on the particle inflight dynamics for the case of the hydrophobic surface. As the excitation frequency increase 64 Hz, the inertial force generated on the particle becomes much larger than the adhesion force. Therefore, the inflight dynamics of the repelled particle becomes independent of the surface wetting characteristics, i.e. hydrophobic or hydrophilic.

The optical transmittance of the hydrophobic and hydrophilic film surfaces is carried out after the dust repelling experiments incorporating the sonic excitations. It is worth to mention that the duration of the experiment is kept 20 s for hydrophobic and hydrophilic surfaces. The relative optical transmittance is evaluated adopting the transmittance ratio, i.e. the relative transmittance (*T*_*rel*_) is: ${T}_{rel}=\frac{{T}_{film}-{T}_{cleaned}}{{T}_{film}}$, here *T*_*film*_ is the optical transmittance of the film, *T*_*cleaned*_ is optical transmittance of the film after the dust is repelled. Table 7 gives the transmittance ratio at 64 Hz of sonic excitation for hydrophobic and hydrophilic film surfaces. The relative transmittance is almost in the order of 57% for hydrophilic surface and it becomes higher (87%) for the hydrophobic surface. The relative transmittance is higher for hydrophobic surface than hydrophilic surface after dust mitigation by sonic excitation. It is worth to mention that the optical transmittance of hydrophobic sample is about 90% of clean (none-dust settled) sample for the wavelength range 600 nm 800 nm^21^. Hence, the use of hydrophobic surface improves the overall optical transmittance of the samples after dust mitigation via sonic excitation. However, a care must be taken to use the hydrophobic coating on the optically transmitting surfaces because of almost 10% reduction in the optical transmittance after hydrophibizing the surface occurs^21^. Hence, hydrophobizing of surfaces lowers the optical transmittances in none-dusty regions; however, it improves overall optical transmittance as the samples surfaces being subjected to regular heavy dust depositions. Moreover, the attainment of a low ratio of optical transmittance is because of the mode of film vibration at 64 Hz, which generates a single-mode and repelled dust particles in the outer region of the film moves towards the film center with multiple excitations during 20 s. Nevertheless, as the film surface tilted more than 30° about the vertical axis, the transmittance ratio is expected to increase significantly.

**Table 7:** Transmittance ratio for hydrophilic and hydrophobic film surfaces after dust repelling.

Surface type	Transmittance ratio
Hydrophilic	0.57
Hydrophobic	0.78

## Conclusion

Dust repelling from transparent polyvinyl chloride film under sonic excitation is examined in relation to dust mitigation from transparent surfaces. An experimental rig is designed and built evaluating the dynamics of the repelling dust particles from the film surface. Environmental dust particles adhesion on hydrophilic and hydrophobic surfaces are evaluated through atomic force microscopy. The film surface is hydrophobized via dip-coating by the functionalized nano-silica particles. High-speed monitoring system and the tracker program are used to measure the vertical and horizontal heights of the infight dust particles for various frequencies of the sonic excitation. The flexural characteristics of the film due to applied sonic power are formulated and the mode of film vibration is determined for different sonic power frequencies. The trajectory of dust particles emanating from the film surface is formulated in a spherical coordinate system and the resulting equations are solved using COMSOL Differential Equation solver. The findings of the dynamic characteristics of the inflight dust particles are compared with those of the experimental counterparts. It is demonstrated that the inflight dust particles heights along the vertical and horizontal directions agree with those of the experimental findings. The film displacement and transverse velocity become the maximum for the excitation frequency of 64 Hz with the standoff distance between the film and the sonic excitation source of 30 mm. In this case, the film vibration results in a single principle vibration mode (0,1), which occurs at the film center. Hydrophobizing the film surface lowers the dust particle adhesion considerably; hence, adhesion force reduces by almost 35% for 0.9 µm particle and about 47% for 8 µm particle. The vertical height of the dust particle is higher for excitation frequency of 64 and reducing the excitation frequency to 45 Hz or increasing to 104 Hz, vertical height reduces significantly. The inflight particle displacement is large in both vertical and horizontal axes, which implies that the particles from the film surface can be repelled away from the film. Hence, sonic excitation can mitigate the dust particles from the transparent polyvinyl chloride surface under a proper selection of the standoff distance and the frequency of the excitation. The hydrophobic surface provides longer repelling distances for dust particles under low (55 Hz) and high (75 Hz) frequency sonic excitations. This is because of low inertia force generated on the dust particle, which is comparable to the force of adhesion of the particle. However, as the sonic excitation frequency is set at 64 Hz, the inertia force generated on the dust particle becomes considerably larger than the adhesion force. Hence, the height of the inflight dust particle does not influence notably with the adhesion force. Dust mitigation by the sonic excitation improves the optical transmittance of the dusty surface while indicating that the sonic excitation can be effectively used for dust removal from the transparent film surfaces.

## Supplementary information

Supplementary Information 1.

Supplementary Information 2.
