# Supplementary material for: Environmental dust repelling from hydrophilic/hydrophobic surfaces under sonic excitations
Source: Sci Rep. 2020 Nov 9;10:19348. doi: 10.1038/s41598-020-76418-2 (PMC7652867; doi:10.1038/s41598-020-76418-2)
Supplement: Supplementary file 1 — Supplementary Information 1. [file 41598_2020_76418_MOESM1_ESM.docx]

Supplementary information

**Environmental Dust Repelling from Hydrophilic/Hydrophobic Surfaces under Sonic Excitations**

**Abba Abdulhamid Abubakar^1^ Bekir Sami Yilbas^1,2,3*^ Hussain Al-Qahtani^1^ Ammar Alzaydi^1^**

^1^Mechanical Engineering Department, King Fahd University of Petroleum and Minerals, Dhahran, 31261, Saudi Arabia

^2^Center of Research Excellence in Renewable Energy (CoRE-RE), KFUPM, Dhahran, 31261, Saudi Arabia

^3^Senior Researcher at K.A. CARE Energy Research & Innovation Center at Dhahran, Saudi Arabia

Correspondence and requests for materials should be addressed to B.S.Y. (Email: bsyilbas@kfupm.edu.sa; Phone: +966 3 860 4481)

**S1: Vibration Analysis of thin film.**

A schematic view of polyvinyl chloride film and the film conditions are shown in Fig. 2 in line with the experiments. The film is very thin (14 µm in thickness) and possesses very negligible flexural stiffness. The transverse deflection of the film ($w_{n}(x,y))$ due to its natural vibration can be obtained from the standard wave equation as expressed in Eq. (1).

$\rho h\frac{\partial^{2}w_{n}(x,y)}{\partial t^{2}}=T_{0}\left( \frac{\partial^{2}w_{n}(x,y)}{\partial x^{2}}+\frac{\partial^{2}w_{n}(x,y)}{\partial y^{2}} \right)$ (1)

Here: $\rho$ is the film density, $t$ is time, $h$ is film thickness, $w_{n} (x,y)$ is transverse deflection, and $T_{0}$ is the in-plane (radial) tensile force per unit length acting at the edges of the film.

Since the film is circular and subjected to nearly uniform radial force per unit length of $T_{0}$ at the edges, using a cylindrical coordinate system, Eq. (1) reduces to:

$\frac{\partial^{2}w_{n}(r,\theta, t)}{\partial t^{2}}=c^{2}\left( \frac{\partial^{2}w_{n}(r,\theta, t)}{\partial r^{2}}+\frac{1}{r}\cdot\frac{\partial w_{n}(r,\theta, t)}{\partial r}+\frac{1}{r^{2}}\cdot\frac{\partial^{2}w_{n}(r,\theta, t)}{\partial\theta^{2}} \right)$ (2)

Here: $c=\sqrt{\frac{T_{0}}{\rho h}}$ is the speed of propagation of the transverse vibrating wave on the film surface.

The following boundary conditions need to be satisfied with zero transverse displacements at the edges, i.e.:

$w_{n}(r,\theta,t)=0$ at $r=R$ (3)

It has been shown that ^1^, the general solution of Eq. (3) can be found by separable of variable as given below:

$w_{n}\left( r,\theta, t \right)=p(r)\cdot q(\theta)\cdot s(t)$ (4)

Substituting Eq. (4) into (3) leads to:

$\frac{s''(t)}{c^{2}s(t)}=\frac{p''(r)}{p(r)}+\frac{p'(r)}{r\cdot p(r)}+\frac{q''(\theta)}{r^{2}\cdot q(\theta)}=K_{1}$ (5)

Since the right-hand side (R.H.S.) is only dependent on $t$ and the left-hand side (L.H.S.) is only dependent on $r$ and $\theta$, Eq. (5) is equal to a negative constant $K_{1}=-\lambda^{2}<0$. Hence, the equation can be split into two as follows:

$s^{''}\left( t \right)=-\lambda^{2}c^{2}s(t)$ (6)

and

$r^{2}\frac{s''(t)}{s(t)}+r^{2}\frac{s''(t)}{s(t)}+\lambda^{2}r^{2}=-\frac{q''(\theta)}{q(\theta)}=K_{2}$ (7)

The solution for Eq. (7) is a linear combination of sine and cosine functions as expressed as:

$s\left( t \right)=A_{1}\cdot\cos\left( c\lambda t \right)+B_{1}\cdot\cos\left( c\lambda t \right)$ (8)

Here: $A_{1}$ and $B_{1}$ are constants and $\omega=c\lambda$ is the angular frequency.

Also, since $-\frac{q''(\theta)}{q(\theta)}=K_{2}$, the solution is a linear combination of sine and cosine functions:

$q\left( \theta\right)=A_{2}\cos\left( m\theta\right)+B_{2}sin(m\theta)$ (9)

Here: $A_{2}$ and $B_{2}$ are constants and $m=\sqrt{K_{2}}=0,1,2,\ldots\ldots$. are constants that represent the number of diametral lines with zero deflection.

For Eq. (7), its solution is a linear combination of Bessel functions, $J_{m}$ and $Y_{m}$ of order 0 as expressed in Eq. (11):

$R\left( r \right)=J_{m}\left( \lambda_{mn}r \right), for m=0,1,\ldots\ldots.., n=1,2,\ldots\ldots..$ (10)

Here: $\lambda_{mn}=\frac{k_{mn}}{R}$ and $k_{mn}$ is the n-th positive root of $J_{m}$.

The closed-form solution to Eq. (4) can be expressed as:

$w_{n}\left( r,\theta, t \right)=\left( A_{1}\cdot\cos\left( c\lambda t \right)+B_{1}\cdot\cos\left( c\lambda t \right) \right)\cdot J_{m}\left( \lambda_{mn}r \right)\cdot\left( A_{2}\cos\left( m\theta\right)+B_{2}sin(m\theta) \right)$ (11)

and the natural frequencies of the vibrating film can be obtained from:

$f_{mn}=c\lambda_{mn}=\frac{c\cdot k_{mn}}{2\pi R}=\frac{k_{mn}}{2\pi R}\sqrt{\frac{T_{0}}{\rho h}}$ (12)

Here: $k_{mn}$ can be obtained from the Bessel function of the first kind, $m$ is an integer that represents the number of circumferential lines with zero deflection and $n$ is an integer that represents the number of diametral lines with zero deflection. Hence, $m$ and $n$ can be used to characterize the mode's shape.

The force vibration of the film becomes necessary because of external sonic excitation from the film bottom. Having known the modes shape, a pulsating (sinusoidal) pressure is applied at the base of the film due to the sound waves emanating from the speaker diaphragm. Generally, the sound is modeled as a pressure wave resulting from the change in pressure from that of ambient. Mathematically, the propagating sine wave can be represented as:

$\Delta P={\Delta P}_{max}\sin(\omega t-kx)$ (13)

where $\Delta P=P-P_{atm}$ is the change in pressure, ${\Delta P}_{max}$ is the amplitude (or maximum) pressure change, $k=\frac{2\pi}{\lambda}$ is the wavenumber, $\omega=\frac{2\pi}{T}=2\pi f$ is the angular frequency, $x$ is spatial coordinate, $t$ is time. Hence, the transverse deflection of the film under forced vibration, $w_{f}(r,\theta, t)$, can be expressed as:

$\rho h\frac{\partial^{2}w_{f}(r,\theta, t)}{\partial t^{2}}=T_{0}\left( \frac{\partial^{2}w_{f}(r,\theta, t)}{\partial r^{2}}+\frac{1}{r}\cdot\frac{\partial w_{f}(r,\theta, t)}{\partial r}+\frac{1}{r^{2}}\cdot\frac{\partial^{2}w_{f}(r,\theta, t)}{\partial\theta^{2}} \right)+\Delta P_{max}sin(2\pi ft)$ (14)

Since the experiment was conducted at low-frequency range in which mode (0,1) dominates, the solution to $w_{f}\left( r,\theta,t \right)$ can be considered to be axisymmetric. Therefore, Eq. (14) can be only a function of $r$ and $\theta$ as expressed as:

$\rho h\frac{\partial^{2}w_{f}(r, t)}{\partial t^{2}}=T_{0}\left( \frac{\partial^{2}w_{f}(r, t)}{\partial r^{2}}+\frac{1}{r}\cdot\frac{\partial w_{f}(r,t)}{\partial r}+\frac{1}{r^{2}}\cdot\frac{\partial^{2}w_{f}(r, t)}{\partial\theta^{2}} \right)+\Delta P_{max}sin(2\pi ft)$ (15)

Before the start of oscillatory motion, the following initial condition needs to be satisfied, i.e.:

$w_{f}\left( R,0 \right)=0 \& \frac{\partial w\left( R,0 \right)}{\partial t}=0 at t=0$ (16)

It has been shown that ^2^, the exact solution of the PDE can be expressed as:

$w_{f}\left( r,t \right)=\frac{\Delta P_{max}c^{2}}{\omega^{2}T_{0}}\sin\left( \omega t \right)\left( \frac{J_{0}\left( \frac{\omega r}{c} \right)}{J_{0}\left( \frac{\omega R}{c} \right)}-1 \right)-\frac{2\Delta P_{max}\omega c}{\alpha T_{0}}\sum_{s=1}^{\infty} \frac{\sin\left( c\alpha_{s}t \right)J_{0}(r\alpha_{s})}{\alpha_{s}^{2}(\omega^{2}-c^{2}\alpha_{s}^{2})J_{0}'(r\alpha_{s})}$ (17)

Here: $s=1,2,\ldots..,$, $\alpha_{s}$ are the roots of $J_{0}\left( az \right)=0$, $I_{0}\left( z \right)=1+\frac{z^{2}}{2^{2}}+\ldots$, $I_{0}\left( iz \right)=J_{0}(z)$, and $I_{0}^{'}\left( iz \right)=-iJ_{0}'(z)$.

**References**

1. Asmar, N. H. *Partial differential equations with Fourier series and boundary value problems*. (Courier Dover Publications, 2016).

2. Carslaw, H. S. & Jaeger, J. C. *Operational methods in applied mathematics*. vol. 123 (Oxford, 1941).
