# Supplementary material for: Environmental dust repelling from hydrophilic/hydrophobic surfaces under sonic excitations
Source: Sci Rep. 2020 Nov 9;10:19348. doi: 10.1038/s41598-020-76418-2 (PMC7652867; doi:10.1038/s41598-020-76418-2)
Supplement: Supplementary file 2 — Supplementary Information 2. [file 41598_2020_76418_MOESM2_ESM.docx]

Supplementary information

**Environmental Dust Repelling from Hydrophilic/Hydrophobic Surfaces under Sonic Excitations**

**Abba Abdulhamid Abubakar^1^ Bekir Sami Yilbas^1,2,3*^ Hussain Al-Qahtani^1^ Ammar Alzaydi^1^**

^1^Mechanical Engineering Department, King Fahd University of Petroleum and Minerals, Dhahran, 31261, Saudi Arabia

^2^Center of Research Excellence in Renewable Energy (CoRE-RE), KFUPM, Dhahran, 31261, Saudi Arabia

^3^Senior Researcher at K.A. CARE Energy Research & Innovation Center at Dhahran, Saudi Arabia

Correspondence and requests for materials should be addressed to B.S.Y. (Email: bsyilbas@kfupm.edu.sa; Phone: +966 3 860 4481)

**S2: Inflight Dust Particle Analysis**

By considering the equilibrium of particle in the spherical coordinate system ($r,\theta,\phi, t$) (Fig. 3), the sum of forces acting on the dust particle at the onset of release can be expressed as:

$\sum F_{r}=m\left( \frac{d^{2}r}{dt^{2}}-r\left( \frac{d\phi}{dt} \right)^{2}\sin^{2} \phi-r\left( \frac{d\theta}{dt} \right)^{2} \right)=-mgcos\theta-D\frac{dr}{dt}+F_{ac}$ (1)

$$\sum F_{\theta}=m\left( 2\frac{dr}{dt}\cdot\frac{d\theta}{dt}+r\frac{d^{2}\theta}{dt^{2}}-r\frac{d^{2}\phi}{dt^{2}}cos\theta\cdot sin\theta\right)=-mgsin\theta cos\phi-D\frac{d\theta}{dt}$$

 (2)

$\sum F_{\phi}=m\left( 2\frac{dr}{dt}\cdot\frac{d\phi}{dt}sin\theta+r\frac{d^{2}\phi}{dt^{2}}sin\theta+2r\frac{d\phi}{dt}\cdot\frac{d\theta}{dt}cos\theta\right)=-mgsin\theta sin\phi-D\frac{d\phi}{dt}$ (3)

Here: $F_{i}$ is the inertia force, $m$ is the mass, $D$ is the drag force term according to Stokes’ law, $d_{p}$ is particle diameter, and $F_{ac}$ is the acoustic force term. Since the flow of air around the dust particle occurs at very low Reynold number $(Re)$, Stokes’ law can be adopted in the drag force terms; thus, $D=3\pi\mu d_{p}$. Also, the acoustic radiation force on a rigid particle moving in a viscous fluid has been previously determined as ^1^: $F_{ac}=\pi\frac{d_{p}^{3}}{8}kfE_{ac}$; here $k$ is the wave number, $E_{ac}=\frac{1}{2\rho_{0}c_{0}^{2}}p_{rms}^{2}$ is acoustic energy density and $k$ is dipole scattering coefficient. The dipole scaterring coefficient is taken as: $f=\frac{6\left( 1-\rho\right)^{2}\left( 1+\delta\right)\delta}{\left( 1+2\rho\right)^{2}+9\left( 1+2\rho\right)\delta+\frac{81}{2\left( \delta^{2}+\delta^{3}+\frac{\delta^{4}}{2} \right)}}$. Here, $\rho=\frac{\rho_{p}}{\rho_{a}}$ is the particle-to-air density ratio and $\delta=\frac{\delta_{b}}{r_{p}}$ is the ratio of boundary layer and particle radius. The boundary layer developed on a spherical particle can be obtained from: $\delta_{b}=\frac{4.53d_{p}}{\sqrt{Re}}$.

Hence, the acceleration of the dust particle can be expressed as:

$\frac{d^{2}r}{dt^{2}}=\left( r\left( \frac{d\theta}{dt} \right)^{2}\sin^{2} \phi+r\left( \frac{d\phi}{dt} \right)^{2} \right)-gcos\theta-\frac{3\pi\mu d_{p}}{m}\frac{dr}{dt}+\frac{\pi d_{p}^{3}kfE_{ac}}{8m}$ (4)

$\frac{d^{2}\theta}{dt^{2}}=-\frac{1}{r}\left( 2\frac{dr}{dt}\cdot\frac{d\theta}{dt}-r\frac{d^{2}\phi}{dt^{2}}cos\theta\cdot sin\theta\right)-\frac{3\pi\mu d_{p}}{mr}\frac{d\theta}{dt}-\frac{gsin\theta cos\phi}{r}$ (5)

$\frac{d^{2}\phi}{dt^{2}}=-\frac{1}{r\cdot\sin\theta}\left( 2\frac{dr}{dt}\cdot\frac{d\phi}{dt}\sin\theta+2r\frac{d\phi}{dt}\cdot\frac{d\theta}{dt}cos\theta\right)-\frac{gsin\theta sin\phi}{rsin\theta}-\frac{3\pi\mu d_{p}}{mrsin\theta}\cdot\frac{d\phi}{dt}$ (6)

The solution of the nonlinear system of equations can be solved using the Backward-Euler finite difference method, incorporating the initial conditions that $r=r_{0}$, $\frac{dr_{0}}{dt}=v_{0}$, $\theta=\theta_{0}$, $\frac{d\theta_{0}}{dt}=0$, $\phi=\phi_{0}$, and $\frac{d\phi_{0}}{dt}=0$. Here, $r_{0}$, $\theta_{0}$ and $\phi_{0}$ are the spherical coordinates that define the initial location of the repelled particle on the membrane surface. The initial radial velocity of the repelled dust particle can be obtained for the impulse relation on the dust particle at the onset of take-off. Hence, $v_{0}$ can be expressed as:

$v_{0}=\frac{ma_{m,i}+F_{ac,i}-F_{adh}-mgcos\theta_{0}}{m}\cdot\Delta t$ (7)

Here: $a_{m,i}$ is the initial acceleration imposed on the particle by the vibrating membrane, $F_{ac,i}$ is the initial acoustic radiation force imposed on the particle, $F_{adh}$ is the particle adhesive force, and $\Delta t$ is the impulse time which is selected as 2.5 ms.

**References**

1. Settnes, M. & Bruus, H. Forces acting on a small particle in an acoustical field in a viscous fluid. *Phys. Rev. E* **85**, 16327 (2012).
